# Efficient Synthesis of the Lewis A Tandem Repeat

**DOI:** 10.3390/molecules21050614

**Published:** 2016-05-11

**Authors:** Daisuke Kobayashi, Akiharu Ueki, Tomoya Yamaji, Kazuya Nagao, Akihiro Imamura, Hiromune Ando, Makoto Kiso, Hideharu Ishida

**Affiliations:** 1 Department of Applied Bioorganic Chemistry, Gifu University, 1-1 Yanagido, Gifu-shi, Gifu 501-1193, Japan; moon_side_blow@yahoo.co.jp (D.K.); gcchann.0213@gmail.com (T.Y.); namemnayo_nameneko@yahoo.co.jp (K.N.); aimamura@gifu-u.ac.jp (A.I.); hando@gifu-u.ac.jp (H.A.); kiso@gifu-u.ac.jp (M.K.); 2 Institute for Integrated Cell-Material Sciences (WPI Program), Kyoto University, Yoshida-ushinomiya-cho, Sakyo-ku, Kyoto-shi, Kyoto 606-8302, Japan; 3 Faculty of Pharmaceutical Sciences, Aomori University, 2-3-1 Kobata, Aomori-shi, Aomori 030-0943, Japan

**Keywords:** Lewis A tandem repeat, convergent synthesis, sugar-binding protein

## Abstract

The convergent synthesis of the Lewis A (Le^a^) tandem repeat is described. The Le^a^ tandem repeat is a carbohydrate ligand for a mannose binding protein that shows potent inhibitory activity against carcinoma growth. The Le^a^ unit, {β-d-Gal-(1→3)-[α-l-Fuc-(1→4)]-β-d-GlcNAc}, was synthesized by stereoselective nitrile-assisted β-galactosylation with the phenyl 3-*O*-allyl-2,4,6-tri-*O*-benzyl-1-thio-β-galactoside, and ether-assisted α-fucosylation with fucosyl (*N*-phenyl)trifluoroacetimidate. This common Le^a^ unit was easily converted to an acceptor and donor in high yields, and the stereoselective assembly of the hexasaccharide and dodecasaccharide as the Le^a^ tandem repeat framework was achieved by 2-trichloroacetamido-assisted β-glycosylation and the (*N*-phenyl)trifluoroacetimidate method.

## 1. Introduction

**Figure 1:**
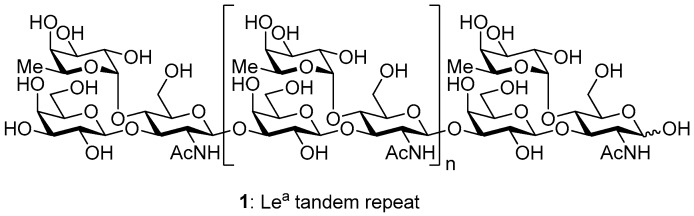
Structure of the Le^a^ tandem repeat.

The Lewis A (Le^a^) trisaccharide, {β-d-Gal-(1→3)-[α-l-Fuc-(1→4)]-β-d-GlcNAc}, is a component of glycolipids that have been identified as antigens of the Lewis blood group. Recently, *N*-linked glycoproteins with Le^a^ tandem repeats (Figure 1) consisting of four or more repeated sequences were isolated from the SW1116 human colorectal carcinoma cell line. This carbohydrate ligand forms a mannose binding protein–carbohydrate complex, which shows potent inhibitory activity against growth of human colorectal carcinoma cells [1,2,3]. To elucidate the inhibitory mechanism, it is necessary to determine and synthesize the minimum structure of the carbohydrate ligand; however, synthesis of the Le^a^ tandem repeat has not been reported. We have recently developed and reported a new synthetic strategy for core 2 decasaccharide with four repeated type-II *N*-acetyllactsamines using a benzyl-protected *N*-trichloroacetyllactsaminyl imidate with high β-selectivity and high yield [4]. In this paper, we describe a synthetic method for the tetrameric Le^a^ tandem repeat motif and the hexasaccharide and dodecasaccharide, via the synthesis of type-I lactosamine by β-selective galactosylation [5] and convergent synthesis with *N*-trichloroacetyllactosaminyl imidate.

## 2. Results and Discussion

**Scheme 1:**
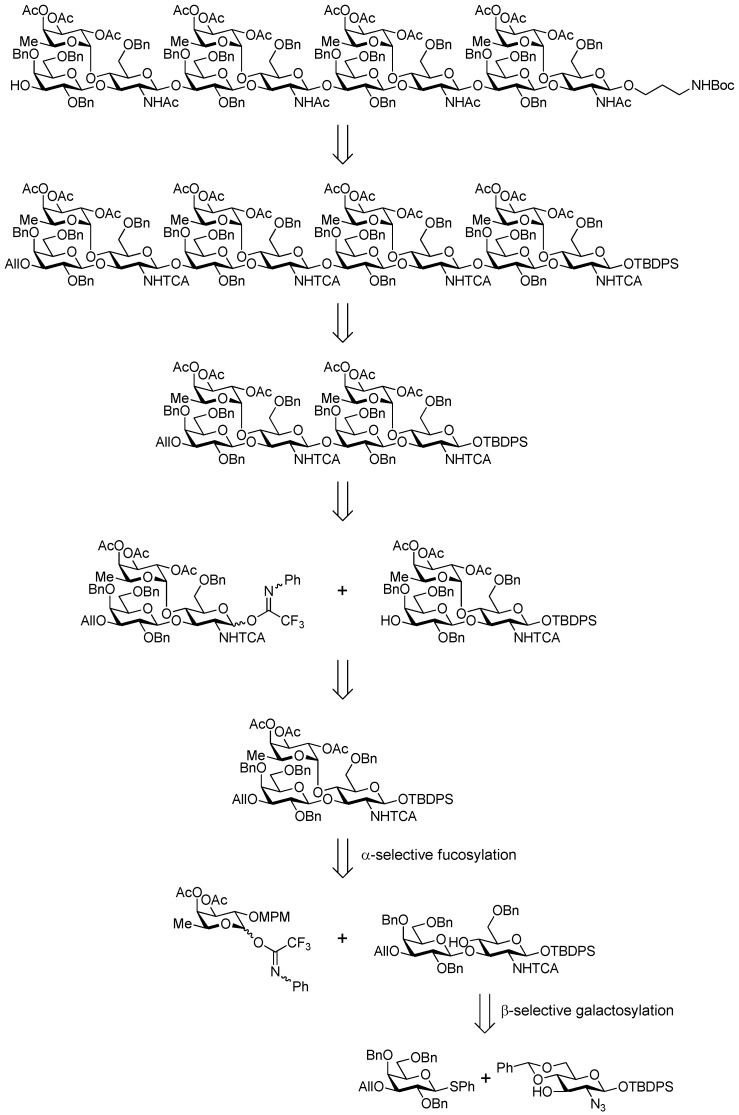
Retrosynthetic scheme.

In the retrosynthetic analysis of the Le^a^ tetramer, we planned the convergent synthesis with a Le^a^ trisaccharide common intermediate, which would be constructed with β-d-Gal-(1→3)-β-d-GlcNTCA synthesized by using β-selective galactosylation and 2-*p*-methoxybenzyl fucosyl imidate. The trisaccharide was designed as the suitably protected form equipped with TBDPS group on the 1-position of the glucosamine and an allyl group on the 3-position of the galactose for divergent synthesis of the acceptor and the donor. In addition, the trichloroacetyl (TCA) group on the 2-position of the glucosamine and the benzyl groups were expected to ensure high stereoselectivity and high yield during the later glycosylation [4,6,7,8,9,10,11,12,13]. We envisioned that the tetramer could be obtained by glycosylation promoted by a catalytic Lewis acid with haxasaccharyl acceptor and donor, which could be prepared from the hexasaccharide Le^a^ dimer, and the hexasaccharide could be synthesized by coupling the acceptor and *N*-phenyl trichloroacetimidyl donor provided from the Le^a^ trisaccharide common intermediate (Scheme 1).

**Scheme 2:**
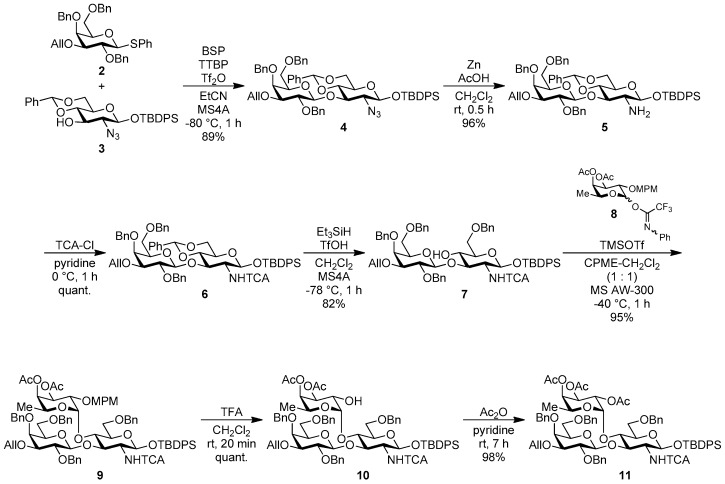
Synthesis of the Le^a^ trisaccharide common intermediate.

Le^a^ trisaccharide **11** was synthesized from galactosyl donor **2** [5], 2-azidoglucosyl acceptor **3** [14], and fucosyl donor **8** [15,16] (Scheme 2). First, disaccharide **4** was constructed from **2** and **3** by propionitrile-mediated β-selective galactosylation [5] under BSP-Tf_2_O-TTBP [17] conditions in 89% yield. Then, **4** was transformed into disaccharide acceptor **7** by reducing the azide group to give **5**, TCA group protection to give **6**, and reductive ring opening of the benzylidene to give **7** in 79% yield (three steps). The glycosylation of **7** with **8** was conducted in cyclopentyl methyl ether-dichloromethane (1:1) [16,18] at −40 °C to obtain trisaccharide **9** in 95% yield. Le^a^ common intermediate **11** was provided by deprotection of a 4-methoxybenzyl group and subsequent acetylation.

**Scheme 3:**
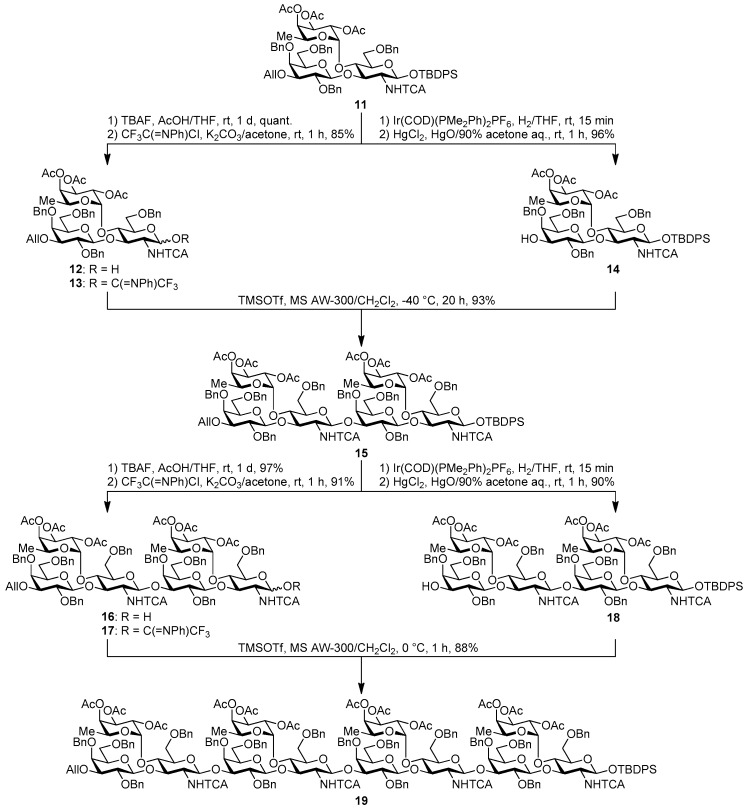
Synthesis of Le^a^ tetramer.

**Scheme 4:**
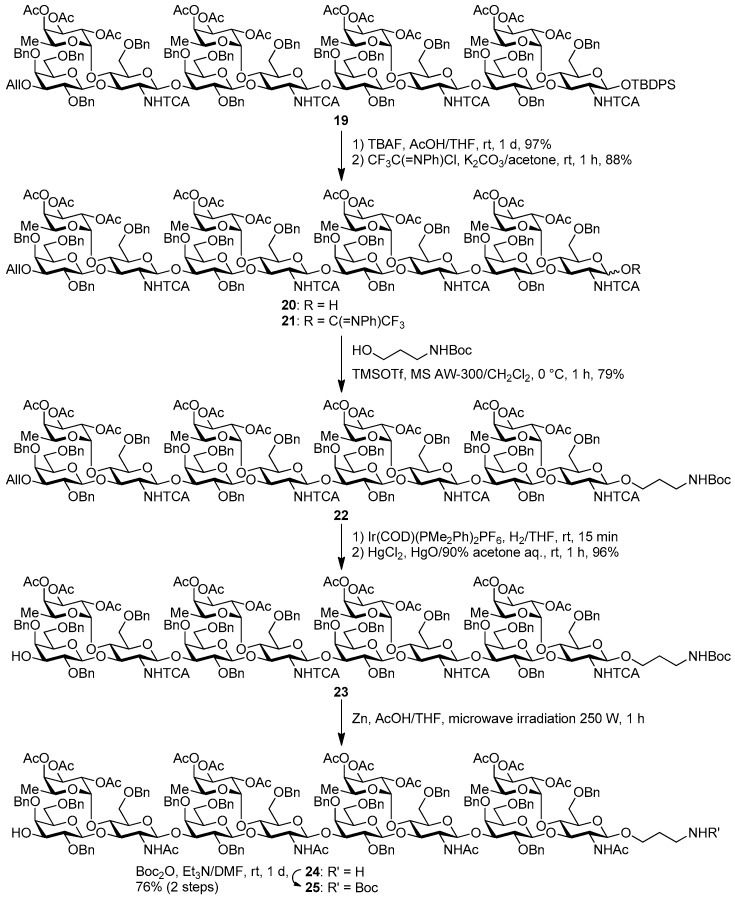
Introduction of a linker to the dodecasaccharide.

Le^a^ trisaccharide **11** was readily converted to donor **13** and acceptor **14** (Scheme 3). After desilylation of **11**, the resulting hemiacetal **12** was treated with (*N*-phenyl)trifluoroacetimidoyl chloride [19] and K_2_CO_3_ to obtain (*N*-phenyl)trifluoroacetimidate **13**. Acceptor **14** was prepared by selective deallylation with iridium-catalyzed olefin migration, followed by treatment with HgCl_2_ and HgO in aqueous acetone solution [20]. The glycosylation of **13** and **14** promoted by catalytic TMSOTf proceeded at −78 °C to give hexasaccharide **15** in 93% yield [4,21,22]. By using the same procedure, hexasaccharide donor **17** and acceptor **18** were prepared in high yields, respectively. In the next coupling, the oligosaccharides showed lower reactivity, and the reaction occurred at 0 °C to afford dodecasaccharide **19** (88%). Next, the linker for sugar probes was introduced (Scheme 4). Dodecasaccharide **19** was converted to (*N*-phenyl)trifluoroacetimidate **21** (85% over two steps) in an analogous manner, and coupled with *N*-Boc aminopropanol to give **22** in 79% yield. After deallylation, microwave-assisted reductive dehalogenation of the TCA group was attempted with excess Zn and AcOH in several solvents (ethyl acetate, 1,4-dioxane, AcOH, and THF) [10]. However, Boc group was cleaved, giving the mixture of aminopropyl and acetamidopropyl derivatives as the main products. It was found that THF was most suitable to obtain aminopropyl derivative **24** almost predominantly. Then, the terminal amino group of **24** was re-protected with Boc group to afford fine **25** in 76% over two steps.

## 3. Experimental Section

### 3.1. General Methods

^1^H- and ^13^C-NMR spectra were recorded with a spectrometer (Avance III 500, Bruker, Billerica, MA, USA). Chemical shifts are expressed in ppm (δ) relative to the Me_4_Si signal as an internal standard. Electrospray ionization time-of-flight high-resolution mass spectrometry was performed (micrOTOF, Bruker Daltonics, Billerica, MA, USA). Specific rotations were determined with a high-sensitivity polarimeter (SEPA-300, Horiba, Kyoto, Japan). Microwave irradiation was carried out in a microwave reactor (μReactor Ex, Shikoku Instrumentation Co., Ltd., Kagawa, Japan). TLC analysis was performed on glass TLC plates (silica gel 60F_254_, Merck, Darmstadt, Germany). Compounds were visualized either by exposure to UV light (254 nm) or by dipping in a solution of 10% H_2_SO_4_ in ethanol, in a solution of phosphomolybdic acid, H_3_PO_4_, and H_2_SO_4_, in H_2_O, or in ninhydrin reagent, followed by heating. Column chromatography was performed with the solvent system (*v*/*v*) specified on silica gel BW-80S, BW-300, PSQ-60B (Fuji Silysia Chemical Ltd. Kasugai, Japan), or Wakosil HC-N (Wako Pure Chemical Industries, Ltd. Osaka, Japan). Gel permeation chromatography was performed with the solvent system (*v*/*v*) specified on Sephadex LH-20 (GE Healthcare UK Ltd. Little Chalfont, UK), or Bio-beads S-X1, S-X3 (Bio-Rad Laboratories, Inc. Hercules, CA, USA). Evaporation and concentration were carried out *in vacuo*.

### 3.2. Physical Data for All New Compounds


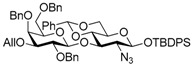


*tert-Butyldiphenylsilyl 3-O-allyl-2,4,6-tri-O-benzyl-β-d-galactopyranosyl-(1→3)-2-azido-4,6-O-benzylidene-2-deoxy-β-d-glucopyranoside* (**4**). To a mixture of phenyl 3-*O*-allyl-2,4,6-tri-*O*-benzyl-1-thio-β-d-galactopyranoside **2** (438 mg, 0.75 mmol), *tert*-butyldiphenylsilyl 2-azido-4,6-*O*-benzylidene-2-deoxy-β-d-glucopyranoside **3** (200 mg, 0.38 mmol), benzenesulfinyl piperidine (237 mg, 1.13 mmol), tri-*tert*-butylpyrimidine (373 mg, 1.50 mmol), and molecular sieves 4A (1.41 g) in propionitrile (12.5 mL) was added dropwise trifluoromethanesulfonic anhydride (140 μL, 0.83 mmol) at −80 °C under Ar, and stirred for 1 h at −80 °C. The reaction mixture was quenched with sat. NaHCO_3_ aq., filtered through Celite, and diluted with EtOAc. The organic layer was separated, and the aqueous layer was extracted with EtOAc. The combined organic layer was successively washed with brine, dried over Na_2_SO_4_, and concentrated. The crude product was chromatographed on silica gel (PSQ-60B) with toluene–acetone (98:2) to give the title product **4** (338 mg, 89%). [α]_D_ −31.3° (*c* 1.1, CHCl_3_); ^1^H-NMR (500 MHz, CDCl_3_) δ 7.70–7.15 (m, 30H, Ar), 5.93–5.85 (m, 1H, H_2_C=CHCH_2_), 5.39 (s, 1H, >CHPh), 5.31–5.27 (m, 1H, H_2_C=CHCH_2_), 5.16–5.13 (m, 1H, H_2_C=CHCH_2_), 4.96–4.89 (m, 2H, PhCH_2_ × 2), 4.80 (d, 1H, *J*_gem_ = 10.7 Hz, PhCH_2_), 4.63 (d, 1H, *J*_1,2_ = 7.9 Hz, H-1^Gal^), 4.57 (d, 1H, *J*_gem_ = 11.7 Hz, PhCH_2_), 4.48 (d, 1H, *J*_1,2_ = 7.8 Hz, H-1^GlcN^), 4.26–4.19 (m, 2H, PhCH_2_ × 2), 4.15–4.09 (m, 2H, H_2_C=CHCH_2_), 3.89 (dd, 1H, *J*_5,6b_ = 4.9 Hz, *J*_gem_ = 10.1 Hz, H-6a^GlcN^), 3.78–3.69 (m, 4H, H-2^Gal^, H-4^Gal^, H-3^GlcN^, H-4^GlcN^), 3.59–3.51 (m, 3H, H-2^GlcN^, H-6b^GlcN^, H-6a^Gal^), 3.36–3.31 (m, 2H, H-3^Gal^, H-6b^Gal^), 3.25 (dd, 1H, *J*_5,6a_ = *J*_5,6b_ = 6.5 Hz, H-5^Gal^), 2.96–2.91 (m, 1H, H-5^GlcN^), 1.16 (s, 9H, ^t^Bu); ^13^C-NMR (125 MHz, CDCl_3_) δ 138.9, 138.8, 137.9, 137.3, 135.8, 134.9, 133.1, 132.5, 130.0, 129.8, 128.9, 128.4, 128.2, 128.2, 128.2, 128.1, 128.0, 127.9, 127.7, 127.6, 127.5, 127.4, 127.4, 126.0, 116.5, 102.7, 101.1, 97.2, 82.3, 79.9, 79.8, 78.7, 75.2, 74.4, 73.5, 73.1, 72.9, 71.7, 68.9, 68.8, 68.3, 66.2, 26.8, 19.1. HRMS (ESI) *m*/*z*: found [M + Na]^+^ 1026.4337, C_59_H_65_N_3_O_10_Si calcd. for [M + Na]^+^ 1026.4337.


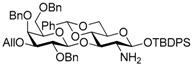


*tert-Butyldiphenylsilyl 3-O-allyl-2,4,6-tri-O-benzyl-β-d-galactopyranosyl-(1→3)-2-amino-4,6-O-benzylidene-2-deoxy-β-d-glucopyranoside* (**5**). A mixture of **4** (576 mg, 0.57 mmol), powdered Zn (1.50 g, 23.0 mmol), and AcOH (0.66 ml, 11.5 mmol) in CH_2_Cl_2_ (14 mL) was stirred for 30 min at room temperature under Ar. The mixture was diluted with CHCl_3_ and filtered through Celite. The filtrate was evaporated, and the residue was diluted with CHCl_3_. The organic layer was successively washed with sat. NaHCO_3_, water, and brine, dried over Na_2_SO_4_, and concentrated. The residue was chromatographed on silica gel with toluene–MeOH (95:5) to give the title product **5** (540 mg, 96%). [α]_D_ −21.8° (*c* 1.3, CHCl_3_); ^1^H-NMR (500 MHz, CDCl_3_) δ 7.68–7.18 (m, 30H, Ar), 5.94–5.87 (m, 1H, H_2_C=CHCH_2_), 5.45 (s, 1H, >CHPh), 5.33–5.28 (m, 1H, H_2_C=CHCH_2_), 5.18–5.15 (m, 1H, H_2_C=CHCH_2_), 4.93–4.88 (m, 2H, PhCH_2_ × 2), 4.78 (d, 1H, *J*_gem_ = 10.9 Hz, PhCH_2_), 4.58 (d, 1H, *J*_gem_ = 11.6 Hz, PhCH_2_), 4.49 (d, 1H, *J*_1,2_ = 7.9 Hz, H-1^Gal^), 4.43 (d, 1H, *J*_1,2_ = 7.8 Hz, H-1^GlcN^), 4.31–4.25 (m, 2H, PhCH_2_ × 2), 4.17–4.08 (m, 2H, H_2_C=CHCH_2_), 3.96 (dd, 1H, *J*_5,6a_ = 4.9 Hz, *J*_gem_ = 10.4 Hz, H-6a^GlcN^), 3.87–3.81 (m, 2H, H-2^Gal^, H-4^Gal^), 3.66–3.58 (m, 3H, H-4^GlcN^, H-6b^GlcN^, H-6a^GlcN^), 3.54 (t, 1H, *J*_2,3_ = *J*_3.4_ = 9.2 Hz, H-3^GlcN^), 3.41–3.37 (m, 3H, H-3^Gal^, H-5^Gal^, H-6b^Gal^), 3.03–2.99 (m, 2H, H-2^GlcN^, H-5^GlcN^), 1.20 (s, 9H, ^t^Bu); ^13^C-NMR (125 MHz, CDCl_3_) δ 138.9, 138.5, 137.9, 137.6, 135.8, 135.8, 134.8, 133.4, 132.9, 129.8, 129.7, 128.6, 128.4, 128.3, 128.3, 128.1, 128.1, 127.8, 127.7, 127.7, 127.5, 127.4, 127.3, 126.1, 116.7, 104.3, 100.8, 99.1, 83.5, 82.7, 79.9, 79.4, 75.7, 74.5, 73.5, 73.1, 73.0, 71.4, 68.4, 68.2, 66.8, 60.1, 27.0, 19.2. HRMS (ESI) *m*/*z*: found [M + Na]^+^ 1000.4432, C_59_H_67_NO_10_Si calcd. for [M + Na]^+^ 1000.4432.


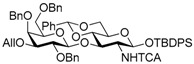


*tert-Butyldiphenylsilyl 3-O-allyl-2,4,6-tri-O-benzyl-β-d-galactopyranosyl-(1→3)-4,6-O-benzylidene-2-deoxy-2-trichloroacetamido-β-d-glucopyranoside* (**6**); To a solution of **5** (12.9 g, 13.2 mmol) in pyridine (132 mL) was added dropwise trichloroacetyl chloride (1.76 mL, 15.8 mmol) at 0 °C under Ar, and stirred at 0 °C for 1 h. The mixture was evaporated, and the residue was diluted with CHCl_3_, successively washed with 2 M HCl, sat. NaHCO_3_, and brine, dried over Na_2_SO_4_, and concentrated. The residue was chromatographed on silica gel with toluene–EtOAc (97:3) to give the title product **6** (14.8 g, quant.). [α]_D_ −9.8° (*c* 1.3, CHCl_3_); ^1^H-NMR (500 MHz, CDCl_3_) δ 7.67–7.18 (m, 30H, Ar), 7.02 (d, 1H, *J*_2,NH_ = 7.0 Hz, NH), 5.95–5.87 (m, 1H, H_2_C=CHCH_2_), 5.45 (s, 1H, >CHPh), 5.34–5.30 (m, 1H, H_2_C=CHCH_2_), 5.24 (d, 1H, *J*_1,2_ = 7.9 Hz, H-1^GlcN^), 5.20–5.17 (m, 1H, H_2_C=CHCH_2_), 4.90 (d, 1H, *J*_gem_ = 11.6 Hz, PhCH_2_), 4.82 (d, 1H, *J*_gem_ = 10.7 Hz, PhCH_2_), 4.69 (d, 1H, PhCH_2_), 4.57 (d, 1H, PhCH_2_), 4.42 (d, 1H, *J*_1,2_ = 7.9 Hz, H-1^Gal^), 4.39–4.30 (m, 3H, PhCH_2_ × 2, H-3^GlcN^), 4.18–4.10 (m, 2H, H_2_C=CHCH_2_), 3.95 (dd, 1H, *J*_5,6a_ = 4.9 Hz, *J*_gem_ = 10.4 Hz, H-6a^GlcN^), 3.82–3.79 (m, 2H, H-2^Gal^, H-4^Gal^), 3.65 (t, 1H, *J*_3,4_ = *J*_4,5_ = 9.2 Hz, H-4^GlcN^), 3.61–3.54 (m, 2H, H-6b^GlcN^, H-6a^Gal^), 3.49 (dd, 1H, *J*_5,6b_ = 5.4 Hz, *J*_gem_ = 9.0 Hz, H-6b^Gal^), 3.44–3.39 (m, 2H, H-2^GlcN^, H-5^Gal^), 3.31 (dd, 1H, *J*_2,3_ = 9.8 Hz, *J*_3,4_ = 2.9 Hz, H-3^Gal^), 3.12–3.07 (m, 1H, H-5^GlcN^), 1.06 (s, 9H, ^t^Bu); ^13^C-NMR (125 MHz, CDCl_3_) δ 161.4, 138.9, 138.7, 137.8, 137.3, 135.9, 135.7, 134.8, 133.0, 132.5, 129.8, 129.8, 128.7, 128.5, 128.4, 128.1, 128.1, 128.0, 127.9, 127.9, 127.8, 127.5, 127.4, 127.4, 126.1, 116.8, 103.1, 100.8, 94.1, 92.2, 82.1, 79.9, 79.2, 77.6, 76.5, 75.8, 74.5, 73.6, 73.3, 73.2, 71.4, 68.3, 68.3, 65.9, 62.7, 26.9, 19.1. HRMS (ESI) *m*/*z*: found [M + Na]^+^ 1144.3368, C_61_H_66_Cl_3_NO_11_Si calcd. for [M + Na]^+^ 1144.3368.


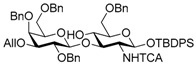


*tert-Butyldiphenylsilyl 3-O-allyl-2,4,6-tri-O-benzyl-β-d-galactopyranosyl-(1→3)-6-O-benzyl-2-deoxy-2-tri-chloroacetamido-β-d-glucopyranoside* (**7**). To a mixture of **6** (814 mg, 0.73 mmol) and molecular sieves 4A (4.20 g) in CH_2_Cl_2_ (7.3 mL) was added triethylsilane (463 μL, 2.90 mmol) and trifluoromethane sulfonic acid (127 μL, 1.45 mmol) at −78 °C under Ar, and stirred for 1 h at −78 °C, and 1.5 h at −40 °C. The reaction mixture was quenched with triethylamine, filtered through Celite, and diluted with CHCl_3_. The organic layer was successively washed with sat. NaHCO_3_, water, and brine, dried over Na_2_SO_4_, and concentrated. The crude product was chromatographed on silica gel with toluene–EtOAc (89:11) to give the title product **7** (665 mg, 82%). [α]_D_ +1.5° (*c* 1.3, CHCl_3_); ^1^H-NMR (500 MHz, CDCl_3_) δ 7.72–7.16 (m, 30H, Ar), 6.70 (d, 1H, *J*_2,NH_ = 7.1 Hz, NH), 5.93–5.86 (m, 1H, H_2_C=CHCH_2_), 5.32–5.29 (m, 1H, H_2_C=CHCH_2_), 5.19–5.17 (m, 1H, H_2_C=CHCH_2_), 5.11 (d, 1H, *J*_1,2_ = 8.0 Hz, H-1^GlcN^), 4.88 (d, 1H, *J*_gem_ = 11.6 Hz, PhCH_2_), 4.82 (d, 1H, *J*_gem_ = 11.3 Hz, PhCH_2_), 4.72 (d, 1H, PhCH_2_), 4.53 (d, 1H, PhCH_2_), 4.42–4.34 (m, 4H, PhCH_2_ × 4), 4.23 (d, 1H, *J*_1,2_ = 7.8 Hz, H-1^Gal^), 4.19–4.12 (m, 2H, H_2_C=CHCH_2_), 4.03 (dd, 1H, *J*_2,3_ = 10.1 Hz, *J*_3,4_ = 8.4 Hz, H-3^GlcN^), 3.80 (d, 1H, *J*_3,4_ = 2.9 Hz, H-4^Gal^), 3.75 (dd, 1H, *J*_2,3_ = 9.8 Hz, H-2^Gal^), 3.69 (s, 1H, OH), 3.58–3.47 (m, 6H, H-4^GlcN^, H-5^Gal^, H-6a^GlcN^, H-6a^Gal^, H-6b^GlcN^, H-6b^Gal^), 3.38–3.33 (m, 1H, H-2^GlcN^), 3.29 (dd, 1 H, H-3^Gal^), 3.17–3.13 (m, 1 H, H-5^GlcN^), 1.06 (s, 9 H, ^t^Bu); ^13^C-NMR (125 MHz, CDCl_3_) δ 161.5, 139.1, 138.6, 138.4, 137.6, 136.0, 135.8, 134.7, 133.1, 132.6, 129.7, 129.7, 128.4, 128.4, 128.2, 128.2, 127.9, 127.8, 127.8, 127.6, 127.5, 127.4, 127.3, 127.3, 116.8, 103.5, 93.7, 92.1, 81.7, 81.1, 79.5, 75.7, 75.1, 74.6, 73.6, 73.5, 73.4, 73.4, 71.7, 69.2, 69.1, 68.3, 61.3, 26.9, 19.1. HRMS (ESI) *m*/*z*: found [M + Na]^+^ 1146.3525, C_61_H_68_Cl_3_NO_11_Si calcd. for [M + Na]^+^ 1146.3525.


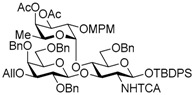


*tert-Butyldiphenylsilyl 3-O-allyl-2,4,6-tri-O-benzyl-β-d-galactopyranosyl-(1→3)-[3,4-di-O-acetyl-6-2-O-p-methoxybenzyl-α-l-fucopyranosyl-(1→4)]-6-O-benzyl-2-deoxy-2-trichloroacetamido-β-d-glucopyranoside* (**9**). To a mixture of **7** (1.96 g, 1.74 mmol), 3,4-di-*O*-acetyl-2-*O*-p-methoxybenzyl-l-fucipyranosyl (*N*-phenyl)-2,2,2-trifluoroacetimidate **8** (1.87 g, 3.47 mmol), and molecular sieves AW-300 (5.22 g) in CPME/CH_2_Cl_2_ (1:1, 58.0 mL) was added TMSOTf (15.7 μL, 0.087 mmol) dropwise at −40 °C under Ar, and stirred for 1 h at −40 °C. The reaction mixture was quenched with sat. NaHCO_3_, filtered through Celite, and diluted with CHCl_3_. The organic layer was separated, and the aqueous layer was extracted with CHCl_3_. The combined organic layer was successively washed with water and brine, dried over Na_2_SO_4_, and concentrated. The crude product was chromatographed on silica gel with hexane–acetone (80:20) to give the title product **9** (2.44 g, 95%). [α]_D_ −19.6° (*c* 1.3, CHCl_3_); ^1^H-NMR (500 MHz, CDCl_3_) δ 7.72–7.11 (m, 32H, Ar), 6.93 (d, 1H, *J*_2,NH_ = 6.8 Hz, NH), 6.81–6.78 (m, 2H, Ar), 5.95–5.87 (m, 1H, H_2_C=CHCH_2_), 5.35–5.32 (m, 1H, H_2_C=CHCH_2_), 5.21–5.11 (m, 5H, H_2_C=CHCH_2_, H-1^GlcN^, H-1^Fuc^, H-3^Fuc^, H-4^Fuc^), 5.09–5.05 (m, 1H, H-5^Fuc^), 4.88 (d, 1H, *J*_gem_ = 10.5 Hz, ArCH_2_), 4.74–4.69 (m, 2H, ArCH_2_ × 2), 4.55–4.49 (m, 3H, ArCH_2_ × 3), 4.46–4.39 (m, 3H, ArCH_2_ × 2, H-1^Gal^), 4.34 (d, 1H, *J*_gem_ = 12.6 Hz, ArCH_2_), 4.24–4.08 (m, 4H, H_2_C=CHCH_2_ × 2, ArCH_2_, H-3^GlcN^), 3.87–3.77 (m, 7H, OMe, H-4^GlcN^, H-4^Gal^, H-6a^Gal^, H-2^Fuc^), 3.73–3.68 (m, 2H, H-6a^GlcN^, H-6b^Gal^), 3.59 (dd, 1H, *J*_1,2_ = 8.0 Hz, *J*_2,3_ = 9.7 Hz, H-2^Gal^), 3.34 (dd, 1H, *J*_5,6a_ = 4.9 Hz, *J*_5,6b_ = 8.9 Hz, H-5^Gal^), 3.30–3.27 (m, 2H, H-2^GlcN^, H-3^Gal^), 3.09 (dd, 1H, *J*_5,6b_ = 1.5 Hz, *J*_gem_ = 11.7 Hz, H-6b^GlcN^), 2.98–2.96 (m, 1H, H-5^GlcN^), 2.11 (s, 3H, Ac), 1.97 (s, 3H, Ac), 1.06 (s, 9H, ^t^Bu), 0.78 (d, 3H, *J*_5,6_ = 6.5 Hz, H-6^Fuc^); ^13^C-NMR (125 MHz, CDCl_3_) δ 170.3, 169.1, 160.9, 159.3, 138.9, 138.5, 138.5, 138.4, 135.8, 135.7, 134.9, 133.4, 132.6, 130.1, 129.7, 129.6, 128.8, 128.5, 128.5, 128.3, 128.3, 128.2, 128.1, 128.0, 127.6, 127.5, 127.4, 127.4, 127.3, 116.8, 113.7, 103.0, 97.1, 93.4, 92.2, 81.7, 79.9, 77.6, 76.0, 75.7, 75.0, 74.6, 73.5, 73.4, 73.3, 73.2, 73.1, 72.6, 72.3, 71.2, 70.8, 67.9, 67.0, 64.2, 55.2, 26.9, 20.9, 20.8, 19.2, 15.4. HRMS (ESI) *m*/*z:* found [M + Na]^+^ 1496.4890, C_79_H_90_Cl_3_NO_18_Si calcd. for [M + Na]^+^ 1496.4890.


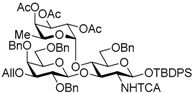


*tert-Butyldiphenylsilyl 3-O-allyl-2,4,6-tri-O-benzyl-β-d-galactopyranosyl-(1→3)-[3,4-di-O-acetyl-α-l-fucopyranosyl-(1→4)]-6-O-benzyl-2-deoxy-2-trichloroacetamido-β-d-glucopyranoside* (**10**). A solution of **9** (29.3 mg, 19.9 μmol) in trifluoroacetic acid/CH_2_Cl_2_ (1:9, 0.80 mL) was stirred for 20 min at room temperature. The reaction mixture was diluted with toluene, and evaporated. The residue was dissolved with CHCl_3_, successively washed with sat. NaHCO_3_, water, and brine, dried over Na_2_SO_4_, and concentrated. The crude product was chromatographed on silica gel with hexane–EtOAC (80:20) to give the title product **10** (27.0 g, quant.). [α]_D_ −33.2° (*c* 1.1, CHCl_3_); ^1^H-NMR (500 MHz, CDCl_3_) δ 7.69–7.15 (m, 30H, Ar), 6.98 (d, 1H, *J*_2,NH_ = 6.7 Hz, NH), 5.97–5.89 (m, 1H, H_2_C=CHCH_2_), 5.36 (dd, 1H, *J*_trans_ = 17.3 Hz, *J*_gem_ = 1.6 Hz, H_2_C=CHCH_2_), 5.23–5.19 (m, 2H, H_2_C=CHCH_2_, H-1^GlcN^), 5.12–5.10 (m, 2H, H-1^Fuc^, H-4^Fuc^), 5.02–4.95 (m, 2H, H-5^Fuc^, H-3^Fuc^), 4.91 (d, 1H, *J*_gem_ = 10.4 Hz, PhCH_2_), 4.75 (d, 1H, *J*_gem_ = 11.1 Hz, PhCH_2_), 4.68 (d, 1H, PhCH_2_), 4.53 (d, 1H, PhCH_2_), 4.47–4.35 (m, 5H, PhCH_2_ × 4, H-1^Gal^), 4.22–4.11 (m, 3H, H-3^GlcN^, H_2_C=CHCH_2_ × 2), 3.92 (t, 1H, *J*_3,4_ = *J*_4,5_ = 9.4 Hz, H-4^GlcN^), 3.85–3.80 (m, 2H, H-2^Fuc^, H-4^Gal^), 3.70–3.56 (m, 4H, H-6a^Gal^, H-6b^Gal^, H-6a^GlcN^, H-2^Gal^), 3.34 (dd, 1H, *J*_5,6a_ = 4.9 Hz, *J*_5,6b_ = 8.7 Hz, H-5^Gal^), 3.30 (dd, 1H, *J*_2,3_ = 9.8 Hz, *J*_3,4_ = 2.8 Hz, H-3^Gal^), 3.22–3.19 (m, 2H, H-6b^GlcN^, H-2^GlcN^), 2.95–2.93 (m, 1H, H-5^GlcN^), 2.13 (s, 3H, Ac), 2.05 (s, 3H, Ac), 1.75 (d, 1H, *J*_2,OH_ = 10.8 Hz, OH), 1.03 (s, 9H, ^t^Bu), 0.81 (d, 3H, *J*_5,6_ = 6.5 Hz, H-6^Fuc^); ^13^C-NMR (125 MHz, CDCl_3_) δ 170.4, 170.2, 161.0, 138.8, 138.4, 138.2, 138.0, 135.9, 135.7, 134.8, 133.2, 132.5, 129.7, 129.7, 128.7, 128.6, 128.5, 128.3, 128.3, 128.2, 128.2, 127.7, 127.6, 127.5, 127.3, 116.9, 103.0, 97.5, 93.2, 92.1, 81.8, 80.1, 77.6, 76.2, 75.9, 74.6, 73.4, 73.3, 73.0, 72.9, 72.4, 71.8, 71.3, 67.9, 67.3, 67.2, 64.6, 63.4, 29.7, 26.9, 21.0, 20.7, 19.2, 15.4. HRMS (ESI) *m*/*z*: found [M + Na]^+^ 1376.4315, C_71_H_82_Cl_3_NO_17_Si calcd. for [M + Na]^+^ 1376.4315.


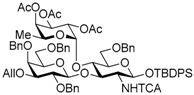


*tert-Butyldiphenylsilyl 3-O-allyl-2,4,6-tri-O-benzyl-β-d-galactopyranosyl-(1→3)-[2,3,4-tri-O-acetyl-α-l-fucopyranosyl-(1→4)]-6-O-benzyl-2-deoxy-2-trichloroacetamido-β-d-glucopyranoside* (**11**). To a solution of **10** (9.32 g, 6.87 mmol) in pyridine (458 mL) was added acetic anhydride (458 mL) at 0 °C under Ar, and stirred for 7 h at room temperature. The reaction mixture was concentrated. The crude product was chromatographed on silica gel with hexane–EtOAc (67:33) to give the title product **11** (9.45 g, 98%). [α]_D_ −37.1° (*c* 1.2, CHCl_3_); ^1^H-NMR (500 MHz, CDCl_3_) δ 7.69–7.14 (m, 30H, Ar), 6.99 (d, 1H, *J*_2,NH_ = 6.7 Hz, NH), 5.97–5.90 (m, 1H, H_2_C=CHCH_2_), 5.35 (dd, 1H, *J*_trans_ = 17.3 Hz, *J*_gem_ = 1.7 Hz, H_2_C=CHCH_2_), 5.22–5.11 (m, 7H, H_2_C=CHCH_2_, H-1^GlcN^, H-1^Fuv^, H-2^Fuc^, H-3^Fuc^, H-4^Fuc^, H-5^Fuc^), 4.91 (d, 1H, *J*_gem_ = 10.3 Hz, PhCH_2_), 4.75 (d, 1H, *J*_gem_ = 11.0 Hz, PhCH_2_), 4.67 (d, 1H, PhCH_2_), 4.53–4.50 (m, 2H, PhCH_2_ × 2), 4.46–4.43 (m, 2H, PhCH_2_, H-1^Gal^), 4.38 (d, 1H, *J*_gem_ = 12.5 Hz, PhCH_2_), 4.31 (d, 1H, PhCH_2_), 4.25 (t, 1H, *J*_2,3_ = *J*_3,4_ = 9.4 Hz, H-3^GlcN^), 4.19–4.11 (m, 2H, H_2_C=CHCH_2_), 3.90 (t, 1H, *J*_4,5_ = 9.4 Hz, H-4^GlcN^), 3.84 (d, 1H, *J*_3,4_ =2.5 Hz, H-4^Gal^), 3.79–3.71 (m, 2H, H-6a^Gal^, H-6b^Gal^), 3.60 (dd, 1H, *J*_1,2_ = 8.0 Hz, *J*_2,3_ = 9.7 Hz, H-2^Gal^), 3.37–3.34 (m, 1H, H-5^Gal^), 3.31 (dd, 1H, H-3^Gal^), 3.24–3.15 (m, 3H, H-2^GlcN^, H-6a^GlcN^, H-6b^GlcN^), 2.99–2.97 (m, 1H, H-5^GlcN^), 2.15 (s, 3H, Ac), 1.97 (s, 3H, Ac), 1.96 (s, 3H, Ac), 1.03 (s, 9 H, ^t^Bu), 0.82 (d, 3H, *J_5,6_* = 6.5 Hz, H-6^Fuc^); ^13^C-NMR (125 MHz, CDCl_3_) δ 170.5, 170.3, 169.3, 160.9, 138.7, 138.5, 138.4, 138.0, 135.9, 135.8, 134.9, 133.3, 132.7, 129.6, 128.7, 128.6, 128.5, 128.3, 128.3, 128.2, 127.6, 127.5, 127.4, 127.4, 127.3, 116.9, 103.1, 95.3, 93.1, 92.1, 81.8, 80.1, 76.2, 75.9, 74.7, 74.5, 73.3, 73.1, 73.1, 72.6, 72.5, 71.7, 71.2, 68.2, 68.1, 68.0, 66.8, 64.3, 63.6, 26.9, 20.8, 20.8, 20.7, 19.2, 15.3. HRMS (ESI) *m*/*z*: found [M + Na]^+^ 1418.4421, C_73_H_84_Cl_3_NO_18_Si calcd. for [M + Na]^+^ 1418.4421.


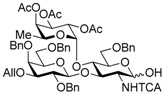


*3-O-Allyl-2,4,6-tri-O-benzyl-β-d-galactopyranosyl-(1→3)-[2,3,4-tri-O-acetyl-α-l-fucopyranosyl-(1→4)]-6-O-benzyl-2-deoxy-2-trichloroacetamido-d-glucopyranose* (**12**). To a solution of **11** (1.67 g, 1.19 mmol) in THF (11.9 mL) were added acetic acid (0.68 mL, 11.9 mmol) and 1 M tetra-n-butylammonium fluoride in THF (4.76 mL, 4.76 mmol) at 0 °C under Ar, and stirred for 1 d at room temperature. The reaction mixture was concentrated. The residue was diluted with EtOAc and water, and extracted with EtOAc. The combined organic layer was successively washed with sat. NaHCO_3_, water, and brine, dried over Na_2_SO_4_, and concentrated. The crude product was purified by silica gel column chromatography with hexane–EtOAc (60:40) and gel permeation chromatography (LH-20, CHCl_3_-MeOH (50:50)) to give the title product **12** (1.38 g, quant.). ^1^H-NMR (500 MHz, CDCl_3_) δ 7.41–7.20 (m, 20H, Ar), 6.76 (d, 1H, *J*_2,NH_ = 9.7 Hz, NH), 5.87–5.80 (m, 1H, H_2_C=CHCH_2_), 5.30–5.12 (m, 7H, H_2_C=CHCH_2_ × 2, H-1^GlcN^, H-1^Fuc^, H-2^Fuc^, H-3^Fuc^, H-4^Fuc^), 4.97 (dd, 1H, *J*_4,5_ = 12.8 Hz, *J*_5,6_ = 6.5 Hz, H-5^Fuc^), 4.87 (d, 1H, *J*_gem_ = 12.1 Hz, PhCH_2_), 4.72 (d, 1H, *J*_gem_ = 11.4 Hz, PhCH_2_), 4.63–4.47 (m, 6H, PhCH_2_ × 5, H-1^Gal^), 4.44 (d, 1H, *J*_gem_ = 11.6 Hz, PhCH_2_), 4.34–4.28 (m, 1H, H-2^GlcN^), 4.19–4.00 (m, 4H, H_2_C=CHCH_2_ × 2, H-3^GlcN^, H-6a^GlcN^), 3.96–3.91 (m, 1H, H-4^GlcN^), 3.81–3.76 (m, 2H, H-4^Gal^, H-6a^Gal^), 3.70 (dd, 1H, *J*_5,6b_ = 7.4 Hz, *J*_gem_ = 9.3 Hz, H-6b^Gal^), 3.64–3.46 (m, 3H, H-2^Gal^, H-5^GlcN^, H-6b^GlcN^), 3.41–3.39 (m, 1H, H-5^Gal^), 3.22 (dd, 1H, *J*_2,3_ = 9.7 Hz, *J*_3,4_ = 2.7 Hz, H-3^Gal^), 3.09–3.08 (m, 1H, OH), 2.13 (s, 3H, Ac), 2.00 (s, 3H, Ac), 1.99 (s, 3H, Ac), 0.75 (d, 3H, H-6^Fuc^); ^13^C-NMR (125 MHz, CDCl_3_) δ 170.5, 170.3, 169.5, 161.3, 139.0, 138.6, 138.3, 137.5, 135.0, 134.7, 129.2, 128.8, 128.5, 128.5, 128.4, 128.3, 128.2, 128.2, 128.1, 127.9, 127.8, 127.8, 127.6, 127.5, 127.1, 116.9, 116.6, 103.8, 95.8, 92.7, 91.2, 81.9, 78.6, 77.6, 74.8, 74.3, 74.2, 73.7, 73.3, 73.2, 72.8, 72.3, 71.7, 71.7, 71.1, 68.7, 68.2, 68.1, 68.1, 67.4, 64.6, 64.5, 55.8, 20.8, 20.8, 20.7, 15.4, 15.4. HRMS (ESI) *m*/*z*: found [M + Na]^+^ 1180.3243, C_57_H_66_Cl_3_NO_18_ calcd. for [M + Na]^+^ 1180.3243.


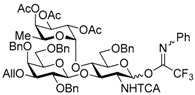


*3-O-Allyl-2,4,6-tri-O-benzyl-β-d-galactopyranosyl-(1→3)-[2,3,4-tri-O-acetyl-α-l-fucopyranosyl-(1→4)]-6-O-benzyl-2-deoxy-2-trichloroacetamido-d-glucopyranosyl (N-phenyl)-2,2,2-trifuoroacetimidate* (**13**). A mixture of **12** (1.35 g, 1.16 mmol), (*N*-phenyl)-2,2,2-trifluoroacetoimidoyl chloride (482 mg, 2.32 mmol), and K_2_CO_3_ (802 mg, 5.80 mmol) in acetone (23.2 mL) was stirred for 1 h at room temperature. The reaction mixture was filtered through Celite, and concentrated. The crude product was purified by gel permeation chromatography [S-X3, toluene–EtOAc (75:25)] and silica gel column chromatography with hexane–EtOAc (71:29) to give the title product **13** (1.31 g, 85%). [α]_D_ −0.7° (*c* 1.4, CHCl_3_); ^1^H-NMR (500 MHz, CDCl_3_) δ 7.38–7.08 (m, 23H, Ar), 6.75–6.71 (m, 3H, Ar, NH), 6.32 (br, 1H, H-1^GlcN^), 5.88–5.80 (m, 1H, H_2_C=CHCH_2_), 5.31–5.21 (m, 5H, H_2_C=CHCH_2_, H-1^Fuc^, H-2^Fuc^, H-3^Fuc^, H-4^Fuc^), 5.14 (dd, 1H, *J*_trans_ = 10.5 Hz, *J*_gem_ = 1.4 Hz, H_2_C=CHCH_2_), 4.95 (dd, 1H, *J*_4,5_ = 10.5 Hz, *J*_5,6_ = 6.4 Hz, H-5^Fuc^), 4.84 (d, 1H, *J*_gem_ = 11.9 Hz, PhCH_2_), 4.73 (d, 1H, *J*_gem_ = 11.4 Hz, PhCH_2_), 4.64–4.49 (m, 7H, PhCH_2_ × 5, H-2^GlcN^, H-1^Gal^), 4.44 (d, 1H, *J*_gem_ = 11.6 Hz, PhCH_2_), 4.16 (t, 1H, *J*_2,3_ = *J*_3,4_ = 9.2 Hz, H-3^GlcN^), 4.10–4.05 (m, 3H, H_2_C=CHCH_2_ × 2, H-4^GlcN^), 3.87–3.81 (m, 3H, H-4^Gal^, H-6a^Gal^, H-5^GlcN^), 3.70 (dd, 1H, *J*_5,6b_ = 7.5 Hz, *J*_gem_ = 9.2 Hz, H-6b^Gal^), 3.62–3.56 (m, 3H, H-2^Gal^, H-6a^GlcN^, H-6b^GlcN^), 3.46–3.44 (m, 1H, H-5^Gal^), 3.24 (dd, 1H, *J*_2,3_ = 9.7 Hz, *J*_3,4_ = 2.7 Hz, H-3^Gal^), 2.14 (s, 3H, Ac), 2.02 (s, 3H, Ac), 2.00 (s, 3H, Ac), 0.78 (d, 3 H, H-6^Fuc^); ^13^C-NMR (125 MHz, CDCl_3_) δ 170.4, 120.2, 169.5, 161.2, 142.9, 138.7, 138.4, 138.2, 137.5, 134.9, 129.2, 129.0, 128.8, 128.5, 128.4, 128.3, 128.2, 128.1, 128.0, 127.8, 127.8, 127.7, 127.6, 127.3, 124.6, 119.3, 116.6, 103.8, 95.9, 92.4, 81.9, 78.5, 77.6, 74.9, 74.8, 74.2, 73.7, 73.7, 73.3, 73.2, 72.2, 71.9, 71.7, 71.6, 68.7, 68.1, 68.0, 66.7, 64.8, 54.7, 20.8, 20.8, 20.7, 15.4. HRMS (ESI) *m*/*z*: found [M + Na]^+^ 1351.3539, C_65_H_70_Cl_3_F_3_N_2_O_18_ calcd. for [M + Na]^+^ 1351.3539.


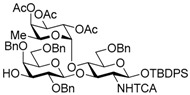


*tert-Butyldiphenylsilyl 2,4,6-tri-O-benzyl-β-d-galactopyranosyl-(1→3)-[2,3,4-tri-O-acetyl-α-l-fucopyranosyl-(1→4)]-6-O-benzyl-2-deoxy-2-trichloroacetamido-β-d-glucopyranoside* (**14**). A mixture of Ir(COD)(PMe_2_Ph)_2_PF_6_ (14.2 mg, 16.8 μmol) in THF (14.0 mL) was stirrede at room temperature for 15 min under H_2_, and the atmosphere was replaced by Ar. To the mixture of activated Ir complex in THF was added a solution of **11** (781 mg, 0.56 mmol) in THF (14.0 mL) under Ar, and stirred for 30 min at room temperature. The reaction mixture was concentrated. The residue was dissolved with 90% acetone aq. (28.0 mL). To the solution were added HgCl_2_ (380 mg, 1.40 mmol) and HgO (48.5 mg, 0.22 mmol), and stirred for 1 h at room temperature. The reaction mixture was diluted with CHCl_3_ and water, and extracted with CHCl_3_. The combined organic layer was successively washed with 10% KI aq., water, and brine, dried over Na_2_SO_4_, and concentrated. The crude product was purified by silica gel column chromatography with hexane–EtOAc (75:25) and gel permeation chromatography (LH-20, CHCl_3_–MeOH (50:50)) to give the title product **14** (727 mg, 96%). [α]_D_ −41.9° (*c* 1.1, CHCl_3_); ^1^H-NMR (500 MHz, CDCl_3_) δ 7.68–7.14 (m, 30H, Ar), 6.85 (d, 1H, *J*_2,NH_ = 7.2 Hz, NH), 5.24–5.18 (m, 4H, H-1^Fuc^, H-2^Fuc^, H-3^Fuc^, H-4^Fuc^), 5.10 (dd, 1H, *J*_4,5_ = 12.9 Hz, *J*_5,6_ = 6.4 Hz, H-5^Fuc^), 5.05 (d, 1H, *J*_1,2_ = 6.7 Hz, H-1^GlcN^), 4.80 (d, 1H, *J*_gem_ = 11.1 Hz, PhCH_2_), 4.76 (d, 1H, PhCH_2_), 4.64 (d, 1H, *J*_gem_ = 11.2 Hz, PhCH_2_), 4.58–4.52 (m, 3H, PhCH_2_ × 3), 4.47 (d, 1H, *J*_1,2_ = 7.8 Hz, H-1^Gal^), 4.38 (d, 1H, *J*_gem_ = 12.5 Hz, PhCH_2_), 4.32 (d, 1H, PhCH_2_), 4.22 (t, 1H, *J*_2,3_ = *J*_3,4_ = 9.3 Hz, H-3^GlcN^), 3.91 (t, 1H, *J*_4,5_ = 9.3 Hz, H-4^GlcN^), 3.83–3.79 (m, 3H, H-4^Gal^, H-6a^Gal^, H-6b^Gal^), 3.51–3.43 (m, 2H, H-3^Gal^, H-5^Gal^), 3.36–3.32 (m, 2H, H-2^GlcN^, H-2^Gal^), 3.25 (dd, 1H, *J*_5,6a_ =2.5 Hz, *J*_gem_ = 11.0 Hz, H-6a^GlcN^), 3.17 (dd, 1H, *J*_5,6b_ = 1.4 Hz, H-6b^GlcN^), 2.98–2.96 (m, 1H, H-5^GlcN^), 2.17 (s, 3H, Ac), 2.10 (d, 1H, *J*_3,OH_ = 6.8 Hz, OH), 1.99 (s, 3H, Ac), 1.96 (s, 3H, Ac), 1.16 (s, 9 H, ^t^Bu), 0.89 (d, 3H, H-6^Fuc^); ^13^C-NMR (125 MHz, CDCl_3_) δ 170.5, 170.2, 169.4, 161.1, 138.8, 138.4, 138.1, 137.9, 135.9, 135.8, 133.2, 132.6, 129.7, 129.7, 128.9, 128.6, 128.5, 128.3, 128.3, 128.3, 128.1, 128.1, 127.9, 127.6, 127.5, 127.5, 127.3, 102.8, 95.4, 93.6, 92.2, 81.2, 77.6, 75.8, 75.4, 75.3, 75.3, 74.6, 74.1, 73.4, 73.2, 73.2, 72.7, 71.7, 68.1, 68.1, 66.8, 64.3, 62.8, 26.9, 20.8, 20.8, 20.8, 19.2, 15.6. HRMS (ESI) *m*/*z*: found [M + Na]^+^ 1378.4108, C_70_H_80_Cl_3_NO_18_Si calcd. for [M + Na]^+^ 1378.4108.


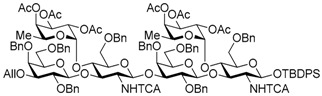


*tert-Butyldiphenylsilyl 3-O-allyl-2,4,6-tri-O-benzyl-β-d-galactopyranosyl-(1→3)-[2,3,4-tri-O-acetyl-α-l-fucopyranosyl-(1→4)]-6-O-benzyl-2-deoxy-2-trichloroacetamido-β-d-glucopyranosyl-(1→3)-2,4,6-tri-O-benzyl-β-d-galactopyranosyl-(1→3)-[2,3,4-tri-O-acetyl-α-l-fucopyranosyl-(1→4)]-6-O-benzyl-2-deoxy-2-trichloroacetamido-β-d-glucopyranoside* (**15**). To a mixture of the glycosyl donor **13** (343 mg, 0.26 mmol), the glycosyl acceptor **14** (234 mg, 0.17 mmol), and molecular sieves AW-300 (516 mg) in CH_2_Cl_2_ (5.7 mL) was added dropwise TMSOTf (3.1 μL, 17.2 μmol) at −40 °C under Ar, and stirred for 20 h at −40 °C. The reaction mixture was quenched with sat. NaHCO_3_, filtered through Celite, and diluted with CHCl_3_. The organic layer was separated, and the aqueous layer was extracted with CHCl_3_. The combined organic layer was successively washed with water and brine, dried over Na_2_SO_4_, and concentrated. The crude product was purified by gel permeation chromatography [S-X1, toluene–EtOAc (75:25)] and silica gel column chromatography with toluene–EtOAc (89:11) to give the title product **15** (399 mg, 93%). [α]_D_ −58.2° (*c* 1.4, CHCl_3_); ^1^H-NMR (500 MHz, CDCl_3_) δ 7.65–6.98 (m, 50H, Ar), 6.62–6.60 (m, 2H, NH × 2), 5.92–5.84 (m, 1H, H_2_C=CHCH_2_), 5.47 (d, 1H, *J*_1,2_ = 7.2 Hz, H-1^GlcN^), 5.32–4.99 (m, 13H, H-1^GlcN^, H-1^Fuc^ × 2, H-2^Fuc^ × 2, H-3^Fuc^ × 2, H-4^Fuc^ × 2, H-5^Fuc^ × 2, H_2_C=CHCH_2_ × 2), 4.78–4.70 (m, 5H, PhCH_2_ × 5), 4.64 (d, 1H, *J*_gem_ = 10.7 Hz, PhCH_2_), 4.60–4.52 (m, 3H, PhCH_2_ × 3), 4.50–4.40 (m, 6H, PhCH_2_ × 5, H-1^Gal^), 4.36–4.32 (m, 2H, PhCH_2_, H-1^Gal^), 4.29–4.17 (m, 3H, PhCH_2_, H-3^GlcN^ × 2), 4.14–4.06 (m, 2H, H_2_C=CHCH_2_ × 2), 4.00 (t, 1H, *J*_3,4_ = *J*_4,5_ = 9.4 Hz, H-4^GlcN^), 3.90 (d, 1H, *J*_3,4_ = 2.3 Hz, H-4^Gal^), 3.85–3.77 (m, 5H, H-4^Gal^, H-4^GlcN^, H-3^Gal^, H-6a^Gal^ × 2), 3.70–3.69 (m, 2H, H-6b^Gal^ × 2), 3.61–3.57 (m, 3H, H-2^Gal^ × 2, H-6a^GlcN^), 3.50–3.38 (m, 5H, H-6b^GlcN^, H-2^GlcN^, H-5^GlcN^, H-5^Gal^ × 2), 3.26 (dd, 1H, *J*_2,3_ = 9.7 Hz, *J*_3,4_ = 2.6 Hz, H-3^Gal^), 3.22 (dd, 1H, *J*_5,6a_ = 2.4 Hz, *J*_gem_ = 11.3 Hz, H-6a^GlcN^), 3.12–3.10 (m, 1H, H-6b^GlcN^), 2.98–2.90 (m, 2H, H-2^GlcN^, H-5^GlcN^), 2.16 (s, 3H, Ac), 2.13 (s, 3H, Ac), 2.08 (s, 3H, Ac), 2.02 (s, 3H, Ac), 1.97 (s, 3H, Ac), 1.93 (s, 3H, Ac), 1.00 (s, 9H, ^t^Bu), 0.79 (d, 3H, *J*_5,6_ = 6.5 Hz, H-6^Fuc^), 0.74 (d, 3H, *J*_5,6_ = 6.5 Hz, H-6^Fuc^); ^13^C-NMR (125 MHz, CDCl_3_) δ 170.5, 170.3, 170.2, 169.4, 169.3, 161.0, 160.8, 139.2, 138.7, 138.5, 138.4, 138.3, 138.0, 137.5, 135.8, 135.7, 134.8, 133.2, 132.6, 129.7, 129.1, 129.0, 128.9, 128.7, 128.4, 128.3, 128.3, 128.2, 128.2, 128.1, 127.9, 127.8, 127.7, 127.6, 127.5, 127.4, 127.3, 116.7, 103.2, 103.2, 95.6, 95.5, 92.2, 92.1, 81.8, 81.2, 79.3, 77.6, 76.1, 75.6, 75.4, 75.2, 75.1, 74.9, 74.6, 74.5, 73.5, 73.3, 73.1, 73.0, 72.9, 72.8, 72.4, 71.7, 71.7, 71.4, 68.2, 68.2, 68.1, 68.0, 66.8, 66.7, 64.5, 64.3, 26.9, 20.9, 20.8, 20.8, 20.7, 19.1, 15.3. HRMS (ESI) *m*/*z*: found [M + Na]^+^ 2517.7349, C_127_H_144_Cl_6_N_2_O_35_Si calcd. for [M + Na]^+^ 2517.7348.


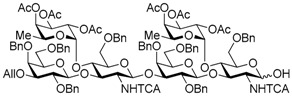


*3-O-Allyl-2,4,6-tri-O-benzyl-β-d-galactopyranosyl-(1→3)-[2,3,4-tri-O-acetyl-α-l-fucopyranosyl-(1→4)]-6-O-benzyl-2-deoxy-2-trichloroacetamido-β-d-glucopyranosyl-(1→3)-2,4,6-tri-O-benzyl-β-d-galactopyranosyl-(1→3)-[2,3,4-tri-O-acetyl-α-l-fucopyranosyl-(1→4)]-6-O-benzyl-2-deoxy-2-trichloroacetamido-d-glucopyranose* (**16**). Compound **15** (453 mg, 0.18 mmol) was desilylated with 1 M TBAF/THF (0.72 mL, 0.72 mmol) and AcOH (0.10 mL, 1.81 mmol) in THF (3.6 mL) as described for **12**. The crude product was purified by gel permeation chromatography [S-X1, toluene–EtOAc (75:25)] and silica gel column chromatography with toluene-EtOAc (67:33) to give the title product **16** (395 mg, 97%). Analysis of compound **16** was too difficult for anomeric isomer, so the product was analyzed in next step. HRMS (ESI) *m*/*z*: found [M + Na]^+^ 2279.6171, C_111_H_126_Cl_6_N_2_O_35_ calcd for [M + Na]^+^ 2279.6170.


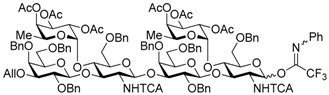


*3-O-Allyl-2,4,6-tri-O-benzyl-β-d-galactopyranosyl-(1→3)-[2,3,4-tri-O-acetyl-α-l-fucopyranosyl-(1→4)]-6-O-benzyl-2-deoxy-2-trichloroacetamido-β-d-glucopyranosyl-(1→3)-2,4,6-tri-O-benzyl-β-d-galactopyranosyl-(1→3)-[2,3,4-tri-O-acetyl-α-l-fucopyranosyl-(1→4)]-6-O-benzyl-2-deoxy-2-trichloroacetamido-d-gluco-pyranosyl (N-phenyl)-2,2,2-trifuoroacetimidate* (**17**). Compound **16** (97 mg, 42.9 μmol) was reacted with (*N*-phenyl)-2,2,2-trifluoroacetoimidoyl chloride (17.8 mg, 85.8 μmol) and K_2_CO_3_ (29.7 mg, 215 μmol) in acetone (1.7 mL) as described for **13**. The crude product was purified by silica gel column chromatography with hexane–EtOAc (83:17) and gel permeation chromatography [S-X1, toluene–EtOAc (75:25)] to give the title product **17** (94.7 mg, 91%). [α]_D_ −35.2° (*c* 1.3, CHCl_3_); ^1^H-NMR (500 MHz, CDCl_3_) δ 7.38–7.07 (m, 43H, Ar), 6.82 (d, 1H, *J*_2,NH_ = 9.4 Hz, NH), 6.71–6.69 (m, 2H, Ar), 6.26 (br, 1H, H-1^GlcN^), 6.00 (d, 1H, *J*_2,NH_ = 4.7 Hz, NH), 5.88–5.81 (m, 1H, H_2_C=CHCH_2_), 5.30–5.14 (m, 10H, H_2_C=CHCH_2_ × 2, H-1^Fuc^ × 2, H-2^Fuc^ × 2, H-3^Fuc^ × 2, H-4^Fuc^ × 2), 5.07 (d, 1H, *J*_1,2_ = 8.1 Hz, H-1^GlcN^), 5.03–5.00 (m, 2H, H-5^Fuc^, PhCH_2_), 4.94 (dd, 1H, *J*_4,5_ = 12.7 Hz, *J*_5,6_ = 6.4 Hz, H-5^Fuc^), 4.78 (d, 1H, *J*_gem_ = 11.6 Hz, PhCH_2_), 4.71 (d, 1H, *J*_gem_ = 11.2 Hz, PhCH_2_), 4.63 (d, 1H, *J*_gem_ = 11.9 Hz, PhCH_2_), 4.60–4.37 (m, 13H, PhCH_2_ ×11, H-1^Gal^, H-2^GlcN^), 4.32 (d, 1H, *J*_1,2_ = 7.8 Hz, H-1^Gal^), 4.20 (t, 1H, *J*_2,3_ = *J*_3,4_ = 9.2 Hz, H-3^GlcN^), 4.07–4.01 (m, 3H, H_2_C=CHCH_2_ × 2, H-4^GlcN^), 3.93–3.77 (m, 8H, H-4^GlcN^, H-2^GlcN^, H-6a^Gal^, H-6a^Gal^, H-4^Gal^, H-5^GlcN^, H-4^Gal^, H-6b^Gal^), 3.76–3.65 (m, 3H, H-3^Gal^, H-3^GlcN^, H-2^Gal^), 3.62–3.52 (m, 5H, H-6a^GlcN^, H-6b^GlcN^, H-6a^GlcN^, H-6b^Gal^, H-2^Gal^), 3.49–3.44 (m, 2H, H-5^Gal^, H-6b^GlcN^), 3.38 (dd, 1H, *J*_5,6a_ = 5.5 Hz, *J*_5,6b_ = 7.5 Hz, H-5^Gal^), 3.21–3.18 (m, 2H, H-3^Gal^, H-5^GlcN^), 2.16 (s, 3H, Ac), 2.15 (s, 3H, Ac), 2.14 (s, 3H, Ac), 2.05 (s, 3H, Ac), 2.01 (s, 6H, Ac × 2), 0.85 (d, 3H, H-6^Fuc^), 0.69 (d, 3H, *J*_5,6_ = 6.4 Hz, H-6^Fuc^); ^13^C-NMR (125 MHz, CDCl_3_) δ 170.4, 170.3, 170.2, 170.1, 169.4, 160.9, 160.7, 142.7, 139.0, 138.8, 138.4, 138.3, 138.2, 138.1, 137.8, 137.6, 137.3, 134.9, 129.3, 129.0, 129.0, 128.8, 128.5, 128.4, 128.4, 128.3, 128.2, 128.2, 128.2, 128.1, 128.1, 127.9, 127.8, 127.7, 127.7, 127.6, 127.5, 127.3, 127.3, 125.2, 124.6, 119.2, 117.0, 116.6, 114.8, 103.4, 103.2, 99.4, 96.0, 95.6, 93.3, 92.6, 92.4, 81.8, 80.8, 78.5, 78.1, 77.6, 76.5, 75.1, 74.9, 74.7, 74.6, 74.4, 74.3, 74.1, 73.4, 73.3, 73.2, 72.9, 72.8, 72.4, 72.0, 71.6, 71.5, 71.4, 68.8, 68.5, 68.2, 68.1, 68.0, 67.9, 67.1, 66.7, 64.8, 64.5, 58.7, 54.6, 30.9, 29.6, 21.4, 20.9, 20.8, 20.8, 20.7, 20.7, 15.7, 15.2, 14.1. HRMS (ESI) *m*/*z*: found [M + Na]^+^ 2450.6466, C_119_H_130_Cl_6_F_3_N_3_O_35_ calcd. for [M + Na]^+^ 2450.6466.


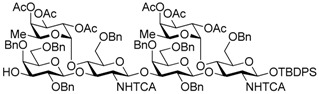


*tert-Butyldiphenylsilyl 2,4,6-tri-O-benzyl-β-d-galactopyranosyl-(1→3)-[2,3,4-tri-O-acetyl-α-l-fucopyranosyl-(1→4)]-6-O-benzyl-2-deoxy-2-trichloroacetamido-β-d-glucopyranosyl-(1→3)-2,4,6-tri-O-benzyl-β-d-galactopyranosyl-(1→3)-[2,3,4-tri-O-acetyl-α-l-fucopyranosyl-(1→4)]-6-O-benzyl-2-deoxy-2-trichloroacetamido-β-d-glucopyranoside* (**18**). Compound **15** (104 mg, 41.6 μmol) was deallylated by Ir(COD)(PMe_2_Ph)_2_PF_6_ (1.1 mg, 1.25 μmol) in THF (1.0 mL × 2) and deprotected by HgCl_2_ (28.2 mg, 104 μmol) and HgO (3.6 mg, 16.6 μmol) with 90% acetone aq. (2.1 mL) as described for **14**. The crude product was purified by silica gel column chromatography with hexane–EtOAc (83:17) and gel permeation chromatography [S-X1, toluene–EtOAc (75:25)] to give the title product **18** (92.2 mg, 90%). [α]_D_ −46.2° (*c* 1.0, CHCl_3_); ^1^H-NMR (500 MHz, CDCl_3_) δ 7.65–7.12 (m, 50H, Ar), 6.63 (d, 1H, *J*_2,NH_ = 7.3 Hz, NH), 6.27 (br, 1H, NH), 5.33–5.08 (m, 10H, H-1^GlcN^, H-1^Fuc^ × 2, H-2^Fuc^ × 2, H-3^Fuc^ × 2, H-4^Fuc^ × 2, H-5^Fuc^), 5.00 (dd, 1H, *J*_4,5_ = 12.3 Hz, *J*_5,6_ = 6.4 Hz, H-5^Fuc^), 4.91 (br, 1H, H-1^GlcN^), 4.84–4.82 (m, 2H, PhCH_2_ × 2), 4.74 (d, 1H, *J*_gem_ = 10.9 Hz, PhCH_2_), 4.67 (d, 1H, *J*_gem_ = 11.3 Hz, PhCH_2_), 4.62–4.53 (m, 6H, PhCH_2_ × 6), 4.51–4.46 (m, 3H, PhCH_2_ × 3), 4.43–4.28 (m, 5H, H-1^Gal^ × 2, PhCH_2_ × 3), 4.15–4.12 (m, 1H, H-3^GlcN^), 4.04–4.00 (m, 2H, H-3^GlcN^, H-4^GlcN^), 3.90–3.87 (m, 2H, H-4^Gal^, H-6a^Gal^), 3.85–3.81 (m, 3H, H-6b^Gal^, H-4^Gal^, H-4^GlcN^), 3.79–3.68 (m, 4H, H-3^Gal^, H-6a^Gal^, H-6b^Gal^, H-2^GlcN^), 3.64–3.60 (m, 2H, H-2^Gal^, H-6a^GlcN^), 3.52 (dd, 1H, *J*_5,6b_ = 2.0 Hz, *J*_gem_ = 10.7 Hz, H-6b^GlcN^), 3.49–3.40 (m, 3H, H-5^Gal^ × 2, H-3^Gal^), 3.36–3.32 (m, 2H, H-5^GlcN^, H-2^Gal^), 3.24–3.12 (m, 3H, H-6^GlcN^ × 2, H-2^GlcN^), 2.92 (d, *J*_4,5_ = 9.6 Hz, H-5^GlcN^), 2.18 (s, 3H, Ac), 2.14 (s, 3H, Ac), 2.09 (s, 3H, Ac), 2.03 (s, 3H, Ac), 2.00 (d, 1H, *J*_3,OH_ = 5.2 Hz, OH), 1.98 (s, 3H, Ac), 1.93 (s, 3H, Ac), 1.00 (s, 9H, ^t^Bu), 0.84 (d, 3H, *J*_5,6_ = 6.4 Hz, H-6^Fuc^), 0.76 (d, 3H, H-6^Fuc^); ^13^C-NMR (125 MHz, CDCl_3_) δ 170.5, 170.3, 170.2, 170.1, 169.5, 169.3, 161.0, 160.7, 139.2, 138.6, 138.5, 138.4, 138.2, 138.1, 137.9, 137.8, 137.4, 135.8, 135.7, 133.1, 132.5, 129.7, 129.6, 129.1, 129.0, 129.0, 128.7, 128.4, 128.4, 128.3, 128.3, 128.2, 128.2, 128.1, 128.0, 128.0, 127.8, 127.8, 127.8, 127.7, 127.6, 127.5, 127.4, 127.4, 127.3, 125.3, 103.1, 99.0, 95.7, 95.5, 93.6, 92.4, 92.3, 81.5, 80.1, 77.6, 77.5, 76.2, 75.3, 75.2, 75.1, 75.1, 75.0, 74.9, 74.9, 74.7, 74.1, 73.9, 73.7, 73.5, 73.1, 73.1, 72.9, 72.9, 72.8, 71.6, 71.6, 68.2, 68.1, 68.1, 68.0, 67.9, 66.8, 66.6, 64.4, 64.3, 62.2, 59.3, 31.9, 29.6, 29.3, 26.8, 26.7, 22.6, 21.4, 20.9, 20.8, 20.8, 20.7, 20.7, 19.1, 18.8, 15.6, 15.4, 14.1. HRMS (ESI) *m*/*z*: found [M + Na]^+^ 2477.7035, C_124_H_140_Cl_6_N_2_O_35_Si calcd. for [M + Na]^+^ 2477.7035.


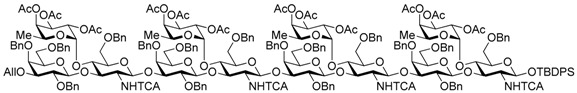


*tert-Butyldiphenylsilyl 3-O-allyl-2,4,6-tri-O-benzyl-β-d-galactopyranosyl-(1→3)-[2,3,4-tri-O-acetyl-α-l-fucopyranosyl-(1→4)]-6-O-benzyl-2-deoxy-2-trichloroacetamido-β-d-glucopyranosyl-(1→3)-2,4,6-tri-O-benzyl-β-d-galactopyranosyl-(1→3)-[2,3,4-tri-O-acetyl-α-l-fucopyranosyl-(1→4)]-6-O-benzyl-2-deoxy-2-trichloroacetamido-β-d-glucopyranosyl-(1→3)-2,4,6-tri-O-benzyl-β-d-galactopyranosyl-(1→3)-[2,3,4-tri-O-acetyl-α-l-fucopyranosyl-(1→4)]-6-O-benzyl-2-deoxy-2-trichloroacetamido-β-d-glucopyranosyl-(1→3)-2,4,6-tri-O-benzyl-β-d-galactopyranosyl-(1→3)-[2,3,4-tri-O-acetyl-α-l-fucopyranosyl-(1→4)]-6-O-benzyl-2-deoxy-2-trichloroacetamido-β-d-glucopyranoside* (**19**). To a mixture of the glycosyl donor **17** (276 mg, 113 μmol), the glycosyl acceptor **18** (210 mg, 85.0 μmol), and molecular sieves AW-300 (255 mg) in CH_2_Cl_2_ (2.8 mL) was added dropwise TMSOTf (3.0 μL, 17.0 μmol) at 0 °C under Ar, and stirred for 1 h at 0 °C. The reaction mixture was quenched with sat. NaHCO_3_, filtered through Celite, and diluted with CHCl_3_. The organic layer was separated, and the aqueous layer was extracted with CHCl_3_. The combined organic layer was successively washed with brine, dried over Na_2_SO_4_, and concentrated. The crude product was purified by gel permeation chromatography [S-X1, toluene–EtOAc (75:25)] and silica gel column chromatography with toluene–EtOAc (83:17) to give the title product **19** (352 mg, 88%). [α]_D_ −50.5° (*c* 1.0, CHCl_3_); ^1^H-NMR (500 MHz, CDCl_3_) δ 7.65–7.02 (m, 90H, Ar), 6.67 (d, 1H, *J*_2,NH_ = 7.1 Hz, NH), 6.27 (brs, 1H, NH), 5.97 (m, 2H, NH × 2), 5.86*-*5.78 (m, 1H, H_2_C=CHCH_2_), 5.43–4.87 (m, 26H, H-1^Fuc^ × 4, H-2^Fuc^ × 4, H-3^Fuc^ × 4, H-4^Fuc^ × 4, H-5^Fuc^ × 4, H-1^GlcN^ × 4, H_2_C=CHCH_2_ × 2), 4.84–4.27 (m, 37H, H-1^Gal^ × 4, H-2^GlcN^, PhCH_2_ × 32), 4.22–3.12 (m, 48H, H-2^Gal^ × 4, H-3^Gal^ × 4, H-4^Gal^ × 4, H-5^Gal^ × 4, H-6a^Gal^ × 4, H-6b^Gal^ × 4, H-2^GlcN^ × 3, H-3^GlcN^ × 4, H-4^GlcN^ × 4, H-5^GlcN^ × 3, H-6a^GlcN^ × 4, H-6b^GlcN^ × 4, H_2_C=CHCH_2_ × 2), 2.93 (d, 1H, *J*_4,5_ = 9.6 Hz, H-5^GlcN^), 2.21–1.88 (m, 36H, Ac × 12), 1.01 (s, 9H, ^t^Bu), 0.89–0.67 (m, 12H, H-6^Fuc^ × 4); ^13^C-NMR (125 MHz, CDCl_3_) δ 171.1, 170.5, 170.4, 170.3, 170.2, 170.2, 169.6, 169.5, 169.4, 160.9, 160.7, 139.4, 139.2, 138.9, 138.6, 138.6, 138.5, 138.5, 138.4, 138.0, 137.9, 137.7, 137.6, 137.5, 136.0, 135.8, 135.0, 133.2, 132.6, 130.0, 129.8, 129.3, 129.2, 129.2, 129.1, 128.8, 128.7, 128.6, 128.5, 128.5, 128.4, 128.4, 128.3, 128.3, 128.2, 128.1, 128.1, 128.0, 127.9, 127.8, 127.8, 127.8, 127.7, 127.6, 127.6, 127.4, 125.4, 116.7, 103.5, 103.3, 103.1, 99.2, 99.1, 98.9, 96.0, 95.9, 95.7, 95.6, 93.7, 92.8, 92.6, 92.4, 81.9, 81.5, 81.4, 78.7, 77.6, 77.4, 76.6, 76.2, 75.6, 75.6, 75.4, 75.3, 75.3, 75.1, 75.0, 75.0, 74.8, 74.5, 74.1, 74.0, 73.9, 73.6, 73.3, 73.3, 73.3, 73.1, 73.0, 73.0, 72.9, 72.6, 72.8, 71.8, 71.7, 68.6, 68.5, 68.3, 68.2, 68.1, 67.0, 66.8, 64.6, 64.5, 64.4, 62.4, 60.4, 59.4, 58.9, 32.0, 29.7, 29.4, 27.0, 22.8, 21.5, 21.1, 21.0, 20.9, 20.9, 20.8, 20.8, 19.2, 15.8, 15.6, 15.4, 14.3, 14.2. HRMS (ESI) *m*/*z*: found [1/2M + Na]^+^ 2369.6540, C_235_H_264_Cl_12_N_4_O_69_Si calcd for [1/2M + Na]^+^ 2369.6544.


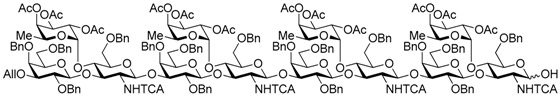


*3-O-Allyl-2,4,6-tri-O-benzyl-β-d-galactopyranosyl-(1→3)-[2,3,4-tri-O-acetyl-α-l-fucopyranosyl-(1→4)]-6-O-benzyl-2-deoxy-2-trichloroacetamido-β-d-glucopyranosyl-(1→3)-2,4,6-tri-O-benzyl-β-d-galactopyranosyl-(1→3)-[2,3,4-tri-O-acetyl-α-l-fucopyranosyl-(1→4)]-6-O-benzyl-2-deoxy-2-trichloroacetamido-β-d-glucopyranosyl-(1→3)-2,4,6-tri-O-benzyl-β-d-galactopyranosyl-(1→3)-[2,3,4-tri-O-acetyl-α-l-fucopyranosyl-(1→4)]-6-O-benzyl-2-deoxy-2-trichloroacetamido-β-d-glucopyranosyl-(1→3)-2,4,6-tri-O-benzyl-β-d-galactopyranosyl-(1→3)-[2,3,4-tri-O-acetyl-α-l-fucopyranosyl-(1→4)]-6-O-benzyl-2-deoxy-2-trichloroacetamido-d-glucopyranose* (**20**). Compound **19** (324 mg, 68.9 μmol) was desilylated with 1 M TBAF/THF (0.28 mL, 0.28 mmol) and AcOH (39.0 μL, 0.69 mmol) in THF (1.4 mL) as described for **12**. The crude product was purified by gel permeation chromatography [S-X1, toluene–EtOAc (75:25)] and silica gel column chromatography with toluene–EtOAc (71:29) to give the title product **20** (298 mg, 97%). [α]_D_ −42.5° (*c* 1.0, CHCl_3_); ^1^H-NMR (500 MHz, CDCl_3_) δ 7.65–7.02 (m, 83H, Ar, NH × 3), 6.70 (d, 1H, *J*_2,NH_ = 9.9 Hz, NH), 5.86–5.79 (m, 1H, H_2_C=CHCH_2_), 5.63–3.11 (m, 112H, H-1^Gal^ × 4, H-2^Gal^ × 4, H-3^Gal^ × 4, H-4^Gal^ × 4, H-5^Gal^ × 4, H-6a^Gal^ × 4, H-6b^Gal^ × 4, H-1^GlcN^ × 4, H-2^GlcN^ × 4, H-3^GlcN^ × 4, H-4^GlcN^ × 4, H-5^GlcN^ × 4, H-6a^GlcN^ × 4, H-6b^GlcN^ × 4, H-1^Fuc^ × 4, H-2^Fuc^ × 4, H-3^Fuc^ × 4, H-4^Fuc^ × 4, H-5^Fuc^ × 4, H_2_C=CHCH_2_ × 2, H_2_C=CHCH_2_ × 2, PhCH_2_ × 32), 2.17–1.99 (m, 36H, Ac × 12), 0.89–0.67 (m, 12H, H-6^Fuc^ × 4); ^13^C-NMR (125 MHz, CDCl_3_) δ 170.4, 170.3, 170.3, 170.3, 170.2, 170.2, 169.5, 169.5, 169.4, 160.7, 160.3, 139.5, 139.5, 139.4, 138.9, 138.5, 138.4, 138.4, 138.4, 138.3, 138.2, 138.1, 137.8, 137.6, 137.5, 137.5, 137.4, 134.9, 129.1, 129.1, 129.0, 128.7, 128.6, 128.5, 128.4, 128.4, 128.4, 128.3, 128.3, 128.2, 128.2, 128.2, 128.1, 128.1, 128.1, 128.0, 127.8, 127.8, 127.7, 127.7, 127.7, 127.7, 127.6, 127.6, 127.6, 127.4, 127.3, 127.3, 127.3, 127.2, 125.3, 116.5, 103.5, 103.4, 103.1, 99.4, 99.3, 99.0, 96.0, 95.8, 95.6, 93.0, 92.9, 92.8, 92.8, 92.6, 91.2, 82.0, 81.8, 81.6, 78.3, 77.6, 76.6, 76.5, 75.7, 75.5, 75.4, 75.3, 75.1, 75.0, 74.8, 74.8, 74.7, 74.7, 74.6, 74.3, 74.2, 74.1, 74.0, 73.4, 73.3, 73.2, 73.2, 73.0, 72.8, 72.8, 72.4, 71.7, 71.6, 71.6, 70.8, 68.6, 68.5, 68.2, 68.1, 68.0, 67.2, 66.9, 66.8, 64.6, 64.4, 57.8, 57.6, 55.6, 29.7, 29.3, 29.3, 21.4, 20.9, 20.9, 20.8, 20.8, 15.8, 15.7, 15.6, 15.5, 15.2, 14.1. HRMS (ESI) *m*/*z*: found [1/2M + Na]^+^ 2250.5955, C_219_H_246_Cl_12_N_4_O_69_ calcd for [1/2M + Na]^+^ 2250.5955.


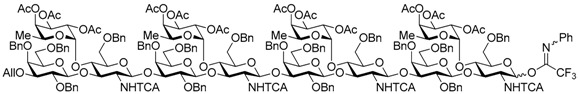


*3-O-Allyl-2,4,6-tri-O-benzyl-β-d-galactopyranosyl-(1→3)-[2,3,4-tri-O-acetyl-α-l-fucopyranosyl-(1→4)]-6-O-benzyl-2-deoxy-2-trichloroacetamido-β-d-glucopyranosyl-(1→3)-2,4,6-tri-O-benzyl-β-d-galactopyranosyl-(1→3)-[2,3,4-tri-O-acetyl-α-l-fucopyranosyl-(1→4)]-6-O-benzyl-2-deoxy-2-trichloroacetamido-β-d-glucopyranosyl-(1→3)-2,4,6-tri-O-benzyl-β-d-galactopyranosyl-(1→3)-[2,3,4-tri-O-acetyl-α-l-fucopyranosyl-(1→4)]-6-O-benzyl-2-deoxy-2-trichloroacetamido-β-d-glucopyranosyl-(1→3)-2,4,6-tri-O-benzyl-β-d-galactopyranosyl-(1→3)-[2,3,4-tri-O-acetyl-α-l-fucopyranosyl-(1→4)]-6-O-benzyl-2-deoxy-2-trichloroacetamido-d-glucopyranosyl (N-phenyl)-2,2,2-trifuoroacetimidate* (**21**). Compound **20** (761 mg, 0.17 mmol) was reacted with (*N*-phenyl)-2,2,2-trifluoroacetoimidoyl chloride (55.0 μL, 0.34 mmol) and K_2_CO_3_ (117 mg, 0.85 mmol) in acetone (6.8 mL) as described for **13**. The crude product was purified by silica gel column chromatography with hexane–EtOAc (83:17) to give the title product **21** (693 mg, 88%). Analysis of compound **21** was too difficult for a few isomers, so the product was analyzed in the next step. ^1^H-NMR of product **21** could not be assigned because all peaks were shown as broad peaks in all range. ^13^C-NMR (125 MHz, CDCl_3_) δ 170.4, 170.3, 170.2, 170.2, 170.2, 170.2, 169.5, 169.5, 160.8, 160.7, 160.4, 142.7, 139.4, 139.3, 139.1, 138.8, 138.5, 138.4, 138.4, 138.3, 138.3, 138.2, 138.1, 137.8, 137.6, 137.5, 137.5, 137.3, 134.9, 129.2, 129.1, 129.0, 129.0, 128.8, 128.6, 128.5, 128.4, 128.4, 128.3, 128.3, 128.2, 128.2, 128.1, 127.9, 127.8, 127.7, 127.6, 127.6, 127.6, 127.6, 127.4, 127.4, 127.3, 127.3, 127.2, 125.3, 124.7, 119.2, 117.0, 116.5, 114.8, 103.5, 103.4, 103.1, 99.3, 99.2, 99.0, 96.1, 95.8, 95.8, 95.6, 93.4, 92.9, 92.7, 92.4, 81.8, 81.6, 78.4, 77.6, 76.6, 76.5, 76.4, 75.6, 75.5, 75.4, 75.3, 75.2, 75.0, 74.8, 74.8, 74.7, 74.3, 74.1, 74.1, 74.0, 73.4, 73.3, 73.2, 73.0, 72.9, 72.9, 72.8, 72.8, 72.4, 72.0, 71.7, 71.6, 71.5, 68.5, 68.1, 68.1, 68.0, 67.9, 67.0, 66.9, 66.8, 66.6, 64.7, 64.5, 64.4, 58.3, 57.9, 54.7, 31.9, 30.9, 29.7, 29.3, 22.7, 21.4, 20.9, 20.9, 20.8, 20.8, 20.7, 15.8, 15.7, 15.6, 15.2, 14.1. HRMS (ESI) *m*/*z*: found [1/2M + Na]^+^ 2336.1103, C_227_H_250_Cl_12_F_3_N_5_O_69_ calcd for [1/2M + Na]^+^ 2329.1103.


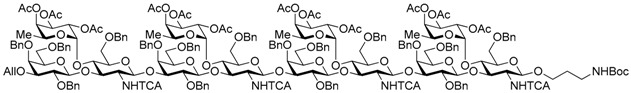


*N-(tert-Butoxycarbonyl)-3-aminopropyl 3-O-allyl-2,4,6-tri-O-benzyl-β-d-galactopyranosyl-(1→3)-[2,3,4-tri-O-acetyl-α-l-fucopyranosyl-(1→4)]-6-O-benzyl-2-deoxy-2-trichloroacetamido-β-d-glucopyranosyl-(1→3)-2,4,6-tri-O-benzyl-β-d-galactopyranosyl-(1→3)-[2,3,4-tri-O-acetyl-α-l-fucopyranosyl-(1→4)]-6-O-benzyl-2-deoxy-2-trichloroacetamido-β-d-glucopyranosyl-(1→3)-2,4,6-tri-O-benzyl-β-d-galactopyranosyl-(1→3)-[2,3,4-tri-O-acetyl-α-l-fucopyranosyl-(1→4)]-6-O-benzyl-2-deoxy-2-trichloroacetamido-β-d-glucopyranosyl-(1→3)-2,4,6-tri-O-benzyl-β-d-galactopyranosyl-(1→3)-[2,3,4-tri-O-acetyl-α-l-fucopyranosyl-(1→4)]-6-O-benzyl-2-deoxy-2-trichloroacetamido-β-d-glucopyranoside* (**22**). To a mixture of the glycosyl donor **21** (144 mg, 31.0 μmol), *N*-Boc-3-amino-1-propanol (53.0 μL, 310 μmol), and molecular sieves AW-300 (93.0 mg) in CH_2_Cl_2_ (1.0 mL) was added dropwise 1% TMSOTf in CH_2_Cl_2_ solution (56.0 μL, 3.1 μmol) at 0 °C under Ar, and stirred for 1 h at 0 °C. The reaction mixture was quenched with sat. NaHCO_3_, filtered by Celite, and diluted with CHCl_3_. The organic layer was separated, and the aqueous layer was extracted with CHCl_3_. The combined organic layer was successively washed with brine, dried over Na_2_SO_4_, and concentrated. The crude product was purified by gel permeation chromatography [S-X1, toluene–EtOAc (75:25)] and silica gel column chromatography with toluene–EtOAc (71:29) to give the title product **22** (113 mg, 79%). [α]_D_ −51.8° (*c* 1.0, CHCl_3_); ^1^H-NMR (500 MHz, CDCl_3_) δ 7.65–7.02 (m, 80H, Ar), 6.67 (d, 1H, *J*_2,NH_ = 6.5 Hz, NH), 6.19 (brs, 1H, NH), 6.02–5.78 (m, 3H, NH × 2, H_2_C=CHCH_2_), 5.31–4.87 (m, 26H, H-1^Fuc^ × 4, H-2^Fuc^ × 4, H-3^Fuc^ × 4, H-4^Fuc^ × 4, H-5^Fuc^ × 4, H-1^GlcN^ × 4, H_2_C=CHCH_2_ × 2), 4.84–4.27 (m, 46H, H-1^Gal^ × 4, H-2^GlcN^ × 3, H-3^GlcN^ × 3, H-4^GlcN^ × 3, PhCH_2_ × 32, NHCH_2_CH_2_CH_2_O), 4.20–3.25 (m, 38H, H-2^Gal^ × 4, H-3^Gal^ × 3, H-4^Gal^ × 4, H-5^Gal^ × 4, H-6a^Gal^ × 4, H-6b^Gal^ × 4, H-3^GlcN^, H-4^GlcN^, H-5^GlcN^ × 3, H-6a^GlcN^ × 3, H-6b^GlcN^ × 3, H_2_C=CHCH_2_ × 2, NHCH_2_CH_2_CH_2_O × 2), 3.27–3.01 (m, 7H, H-3^Gal^, H-2^GlcN^, H-5^GlcN^, H-6^GlcN^ × 2, NHCH_2_CH_2_CH_2_O × 2), 2.21–1.95 (m, 36H, Ac × 12), 1.71–1.59 (m, 2H, NHCH_2_CH_2_CH_2_O × 2), 1.42 (s, 9H, ^t^Bu), 0.84–0.67 (m, 12H, H-6 ^Fuc^ × 4). ^13^C-NMR (125 MHz, CDCl_3_) δ 170.4, 170.3, 170.3, 170.2, 169.5, 169.5, 169.4, 161.2, 160.8, 160.7, 160.6, 156.0, 139.3, 139.1, 139.0, 138.9, 138.5, 138.5, 138.5, 138.4, 138.4, 138.4, 138.3, 137.7, 137.5, 137.5, 135.0, 129.2, 129.1, 129.0, 128.8, 128.6, 128.5, 128.5, 128.4, 128.3, 128.3, 128.2, 128.2, 128.2, 128.2, 128.1, 128.1, 128.0, 127.8, 127.8, 127.8, 127.7, 127.7, 127.7, 127.6, 127.5, 127.4, 116.6, 103.5, 103.1, 99.1, 98.5, 95.9, 95.9, 95.7, 92.7, 92.5, 92.3, 81.9, 81.3, 81.2, 79.2, 78.7, 77.9, 77.6, 76.5, 76.2, 75.5, 75.3, 75.2, 75.1, 75.1, 75.0, 74.9, 74.9, 74.4, 73.9, 73.8, 73.5, 73.3, 73.2, 73.2, 73.1, 73.0, 72.9, 72.5, 71.8, 71.7, 71.6, 68.5, 68.4, 68.3, 68.2, 68.1, 68.1, 67.3, 66.8, 64.6, 64.5, 37.3, 29.7, 28.5, 20.9, 20.8, 20.8, 20.8, 20.8, 15.7, 15.7, 15.5, 15.3. HRMS (ESI) *m*/*z*: found [1/2M + Na]^+^ 2329.1507, C_227_H_261_Cl_12_N_5_O_71_ calcd. for [1/2M + Na]^+^ 2329.1506.


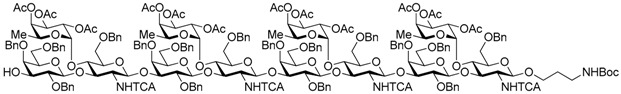


*N-(tert-Butoxycarbonyl)-3-aminopropyl 2,4,6-tri-O-benzyl-β-d-galactopyranosyl-(1→3)-[2,3,4-tri-O-acetyl-α-l-fucopyranosyl-(1→4)]-6-O-benzyl-2-deoxy-2-trichloroacetamido-β-d-glucopyranosyl-(1→3)-2,4,6-tri-O-benzyl-β-d-galactopyranosyl-(1→3)-[2,3,4-tri-O-acetyl-α-l-fucopyranosyl-(1→4)]-6-O-benzyl-2-deoxy-2-trichloroacetamido-β-d-glucopyranosyl-(1→3)-2,4,6-tri-O-benzyl-β-d-galactopyranosyl-(1→3)-[2,3,4-tri-O-acetyl-α-l-fucopyranosyl-(1→4)]-6-O-benzyl-2-deoxy-2-trichloroacetamido-β-d-glucopyranosyl-(1→3)-2,4,6-tri-O-benzyl-β-d-galactopyranosyl-(1→3)-[2,3,4-tri-O-acetyl-α-l-fucopyranosyl-(1→4)]-6-O-benzyl-2-deoxy-2-trichloroacetamido-β-d-glucopyranoside* (**23**). Compound **22** (231 mg, 50.0 μmol) was deallylated by Ir(COD)(PMe_2_Ph)_2_PF_6_ (1.3 mg, 1.50 μmol) in THF (2.5 mL × 2) and deprotected by HgCl_2_ (34.0 mg, 125 μmol) and HgO (4.3 mg, 20.0 μmol) with 90% acetone aq. (5.0 mL) as described for **14**. The crude product was purified by silica gel column chromatography with hexane–EtOAc (75:25) to give the title product **23** (219 mg, 96%). [α]_D_ −59.0° (*c* 1.0, CHCl_3_); ^1^H-NMR (500 MHz, CDCl_3_) δ 7.65–7.02 (m, 80H, Ar), 6.82 (d, 1H, *J*_2,NH_ = 7.0 Hz, NH), 6.22 (brs, 1H, NH), 5.78–5.63 (m, 2H, NH × 2), 5.31–4.88 (m, 23H, H-1^Fuc^ × 4, H-2^Fuc^ × 4, H-3^Fuc^ × 4, H-4^Fuc^ × 4, H-5^Fuc^ × 4, H-1^GlcN^ × 4), 4.87–4.27 (m, 38 H, H-1^Gal^ × 4, H-1^GlcN^, PhCH_2_ × 32, NHCH_2_CH_2_CH_2_O), 4.20 (m, 1H, H-3^GlcN^), 4.03–3.02 (m, 52H, H-2^Gal^ × 4, H-3^Gal^ × 4, H-4^Gal^ × 4, H-5^Gal^ × 4, H-6a^Gal^ × 4, H-6b^Gal^ × 4, H-2^GlcN^ × 4, H-3^GlcN^ × 3, H-4^GlcN^ × 4, H-5^GlcN^ × 4, H-6a^GlcN^ × 4, H-6b^GlcN^ × 4, NHCH_2_CH_2_CH_2_O × 2, NHCH_2_CH_2_CH_2_O × 2), 2.21–1.95 (m, 37H, Ac × 12, OH), 1.71–1.59 (m, 2H, NHCH_2_CH_2_CH_2_O × 2), 1.42 (s, 9H, ^t^Bu), 0.90–0.73 (m, 12H, H-6^Fuc^ × 4); ^13^C-NMR (125 MHz, CDCl_3_) δ 170.5, 170.3, 170.3, 170.2, 170.2, 169.6, 169.6, 169.5, 169.4, 167.8 161.3, 161.0, 160.7, 160.5, 156.0, 139.5, 139., 139.0, 138.9, 138.6, 138.5, 138.5, 138.4, 138.3, 138.3, 138.3, 137.9, 137.7, 137.7, 137.6, 137.5, 132.5, 130.9, 129.2, 129.2, 129.1, 129.0, 128.8, 128.8, 128.6, 128.6, 128.5, 128.5, 128.4, 128.4, 128.3, 128.3, 128.3, 128.2, 128.2, 128.1, 128.1, 127.8, 127.8, 127.8, 127.8, 127.7, 127.7, 127.6, 127.5, 127.4, 125.3, 103.3, 103.0, 99.6, 99.2, 98.6, 95.9, 95.8, 95.7, 92.8, 92.7, 92.4, 81.9, 81.5, 81.3, 79.6, 79.2, 77.9, 76.3, 76.1, 75.5, 75.3, 75.2, 75.2, 75.0, 74.9, 74.7, 74.2, 74.1, 73.8, 73.8, 73.3, 73.3, 73.2, 73.2, 73.1, 73.0, 72.9, 71.7, 71.6, 68.7, 68.6, 68.6, 68.2, 68.1, 67.3, 66.8, 64.6, 64.5, 38.8, 37.2, 30.4, 29.7, 29.0, 28.5, 23.8, 23.0, 21.5, 21.0, 20.9, 20.9, 20.9, 20.8, 20.8, 15.8, 15.6, 14.1, 14.1. HRMS (ESI) *m*/*z*: found [1/2M + Na]^+^ 2309.1352, C_224_H_257_Cl_12_N_5_O_71_ calcd. for [1/2M + Na]^+^ 2309.1350.


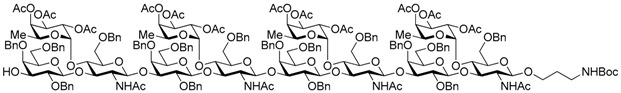


*N-(tert-Butoxycarbonyl)-3-aminopropyl 2,4,6-tri-O-benzyl-β-d-galactopyranosyl-(1→3)-[2,3,4-tri-O-acetyl-α-l-fucopyranosyl-(1→4)]-2-acetamido-6-O-benzyl-2-deoxy-β-d-glucopyranosyl-(1→3)-2,4,6-tri-O-benzyl-β-d-galactopyranosyl-(1→3)-[2,3,4-tri-O-acetyl-α-l-fucopyranosyl-(1→4)]-2-acetamido-6-O-benzyl-2-deoxy-β-d-glucopyranosyl-(1→3)-2,4,6-tri-O-benzyl-β-d-galactopyranosyl-(1→3)-[2,3,4-tri-O-acetyl-α-l-fucopyranosyl-(1→4)]-2-acetamido-6-O-benzyl-2-deoxy-β-d-glucopyranosyl-(1→3)-2,4,6-tri-O-benzyl-β-d-galactopyranosyl-(1→3)-[2,3,4-tri-O-acetyl-α-l-fucopyranosyl-(1→4)]-2-acetamido-6-O-benzyl-2-deoxy-β-d-glucopyranoside* (**25**). A mixture of **23** (60.0 mg, 13.1 μmol), powdered Zn (1.71 g, 26.2 mmol), and AcOH (1.89 mL, 32.8 mmol) in THF (1.3 mL) was stirred under microwave irradiation at 250 W for 1 h under Ar. The mixture was diluted with CHCl_3_ and filtered through Celite. The filtrate was successively washed with sat. NaHCO_3_, water, and brine, dried over Na_2_SO_4_, and concentrated. The residue was chromatographed on silica gel with CHCl_3_–MeOH (92:8). The crude product was dissolved in DMF (1.3 mL), and stirred with Boc_2_O (4.3 μL, 20.0 μmol) and Et_3_N (5.4 μL, 39.0 μmol) at room temperature for 1 d. The reaction mixture was concentrated, and purified by gel permeation chromatography [S-X1, toluene–EtOAc (75:25)] and silica gel column chromatography with CHCl_3_–acetone (80:20–67:33) to give the title product **25** (41.0 mg, 76% in 2steps). ^1^H-NMR of the product **25** could not be assigned because all peaks were shown as broad peaks in all range. [α]_D_ −6.5° (*c* 1.0, CHCl_3_); ^13^C-NMR (125 MHz, CDCl_3_) δ 170.6, 170.4, 170.3, 170.2, 169.5, 169.4, 169.4, 156.0, 139.1, 139.0, 138.8, 138.7, 138.6, 138.6, 138.2, 137.9, 137.8, 137.7, 137.6, 135.9, 129.4, 129.2, 129.1, 129.0, 129.0, 129.0, 128.7, 128.6, 128.6, 128.5, 128.4, 128.3, 128.2, 128.2, 128.2, 128.1, 128.1, 128.0, 128.0, 127.9, 127.7, 127.7, 127.6, 127.6, 127.6, 127.4, 127.3, 127.1, 127.1, 125.3, 103.4, 103.3, 103.2, 100.9, 100.5, 99.0, 95.8, 95.5, 80.5, 79.8, 79.3, 77.6, 76.4, 75.4, 75.2, 75.0, 74.7, 74.6, 74.3, 73.3, 73.3, 73.2, 73.1, 71.8, 68.2, 67.6, 66.7, 64.4, 37.1, 33.7, 32.8, 31.9, 30.2, 30.1, 29.7, 29.5, 29.4, 28.5, 27.1, 23.4, 23.2, 23.2, 22.7, 21.5, 20.8, 20.8, 15.6, 15.5, 15.4, 15.3, 14.1. HRMS (ESI) *m*/*z*: found [1/2M + Na]^+^ 2105.3693, C_224_H_269_N_5_O_71_ calcd for [1/2M + Na]^+^ 2105.3688.

## 4. Conclusions

We have developed a convenient synthesis of the fourth repeated Le^a^ tandem repeat framework. Le^a^ trisaccharide was synthesized by β-selective galactosylation and α-selective fucosylation with high selectivity. Glycosyl acceptors and donors of Le^a^ derivatives were obtained readily in a few steps, and the Le^a^ tandem repeat derivatives, the hexasaccharide and dodecasaccharide, were constructed in high yields. This convergent synthetic strategy can efficiently produce oligosaccharides with repeating structures, which will make an important contribution to biological studies.
